# From Genome to Field—Observation of the Multimodal Nematicidal and Plant Growth-Promoting Effects of *Bacillus firmus* I-1582 on Tomatoes Using Hyperspectral Remote Sensing

**DOI:** 10.3390/plants9050592

**Published:** 2020-05-06

**Authors:** Nik Susič, Uroš Žibrat, Lovro Sinkovič, Andrej Vončina, Jaka Razinger, Matej Knapič, Aleš Sedlar, Saša Širca, Barbara Gerič Stare

**Affiliations:** 1 Plant Protection Department, Agricultural Institute of Slovenia, SI-1000 Ljubljana, Slovenia; nik.susic@kis.si (N.S.); uros.zibrat@kis.si (U.Ž.); andrej.voncina@kis.si (A.V.); jaka.razinger@kis.si (J.R.); matej.knapic@kis.si (M.K.); sasa.sirca@kis.si (S.Š.); 2 Crop Science Department, Agricultural Institute of Slovenia, SI-1000 Ljubljana, Slovenia; lovro.sinkovic@kis.si (L.S.); ales.sedlar@gmail.com (A.S.)

**Keywords:** *Bacillus firmus*, root-knot nematodes, hyperspectral imaging, plant growth promotion, biological pest control

## Abstract

Root-knot nematodes are considered the most important group of plant-parasitic nematodes due to their wide range of plant hosts and subsequent role in yield losses in agricultural production systems. Chemical nematicides are the primary control method, but ecotoxicity issues with some compounds has led to their phasing-out and consequential development of new control strategies, including biological control. We evaluated the nematicidal activity of *Bacillus firmus* I-1582 in pot and microplot experiments against *Meloidogyne luci*. I-1582 reduced nematode counts by 51% and 53% compared to the untreated control in pot and microplot experiments, respectively. I-1582 presence in the rhizosphere had concurrent nematicidal and plant growth-promoting effects, measured using plant morphology, relative chlorophyll content, elemental composition and hyperspectral imaging. Hyperspectral imaging in the 400–2500 nm spectral range and supervised classification using partial least squares support vector machines successfully differentiated *B. firmus*-treated and untreated plants, with 97.4% and 96.3% accuracy in pot and microplot experiments, respectively. Visible and shortwave infrared spectral regions associated with chlorophyll, N–H and C–N stretches in proteins were most relevant for treatment discrimination. This study shows the ability of hyperspectral imaging to rapidly assess the success of biological measures for pest control.

## 1. Introduction

Plant-parasitic nematodes (PPNs) are pervasive pests of a multitude of economically important crop plants, with an impact resulting in economic damage of over $100 billion a year due to yield losses. Of these, root-knot nematodes (RKNs; *Meloidogyne* spp.) are the most damaging PPNs as they can infest a broad range of horticultural and agricultural crop plants [1,2]. *Meloidogyne* spp. (Nematoda: Tylenchida: Meloidogynidae) are soil-borne pathogens infesting roots and causing deformations—the characteristic root galls, limiting the uptake of water and nutrients by the plant and thus causing plant stress [3]. Furthermore, some RKN species, such as *Meloidogyne luci*, have an unresolved taxonomic status, which can hinder official regulatory action against this pest [4,5]. Chemical nematicides are usually the principal control option against PPNs. Historically, fumigant, carbamate or organophosphate nematicides have been most widely used. However, they often pose unacceptable environmental and human health concerns [6]. Many older nematicide types are being replaced by a newer generation of chemicals like fluopyram, secondary metabolites from bacteria, such as avermectins, and various biological agents. Microbiological bionematicides include various fungi (*Pochonia chlamydosporium*, *Myrothecium verrucaria*, *Purpureocillium lilacinus*, *Trichoderma* spp. and *Metarhizium* spp.) and bacteria (*Pasteuria* spp. and *Bacillus* spp.). Such bionematicides also represent an important facet of environmentally conscious strategies for pest management. Of these, rhizobacteria *Bacillus firmus* (Bacillales: Bacillaceae) are one of the most widely used control agents in bionematicide preparations [7,8].

Apart from being utilised in the agricultural sector for crop protection, *B. firmus* is gaining importance for biotechnological applications, such as the production of bioactive compounds, food and feed additives, and expression of industrial enzymes [9]. It has been suggested that *B. firmus’* nematicidal properties are due to various secondary metabolites, enzymes and toxins causing nematode mortality or suppressing nematode reproduction, egg-hatching and juvenile survival [7,10,11,12]. *Bacillus* proteinases, however, are the only nematode-virulence factor to have been confirmed experimentally [10,11]. Isolated from cultivated soil in Israel, isolate *B. firmus* I-1582 has been used commercially as a bionematicide since the early 2000s. Strain I-1582 significantly reduced infestation of different RKN species on tomatoes (*Solanum lycopersicum* L.) and cucumbers (*Cucumis sativus* L.) in pot, greenhouse and field experiments [13,14,15,16]; it induced paralysis and mortality of the RKN *M. incognita*, as well as the burrowing nematode *Radopholus similis* and stem nematode *Ditylenchus dipsaci* [17], and reduced sting nematode *Belonolaimus longicaudatus* infestation on bermudagrass (*Cynodon* spp.) [18], while at the same time increasing plant height and yield [19], and also showing the ability of I-1582 to promote plant growth. Many *Bacillus* spp. have previously been observed to promote plant growth [20]. *B. firmus* bacteria were shown to induce biochemical changes in nematode-parasitised eggplant (*Solanum melongena* L.), aiding stress alleviation [21], while *B. firmus* SW5 was shown to alleviate salt-induced stress in soybean (*Glycine max* L.) in various ways, including the promotion of biomass yield, chlorophyll synthesis and nutrient uptake, as well as influencing the antioxidant defence systems and stress-responsive genes [22].

Plant growth-promoting rhizobacteria (PGPR), such as various bacilli, are rhizosphere-inhabiting bacteria associated with plant roots. PGPR promote plant growth through direct and/or indirect mechanisms. Direct mechanisms include the production and/or metabolism of plant growth regulators (plant hormones) by PGPR and facilitating the uptake of nutrients to the plant (nitrogen fixation and phosphate solubilisation). Indirect mechanisms include the production of siderophores, pathogen-inhibiting factors (cell-wall degrading enzymes and antibiotics) and induced systemic resistance (ISR), eliciting plant host defences by PGPR [20]. The multimodal action of some PGPR—pest control and the promotion of plant growth—can be harnessed in the context of sustainable, environmentally friendly agricultural practices. Apart from a decreased need for chemical pesticides, PGPR can promote more efficient and improved nutrient uptake to crop plants, thus enabling enhanced fertiliser use in agricultural production systems. The long-term overuse of chemical fertilisers to improve productivity often leads to wider negative environmental impact due to nitrate leaching into ground water, phosphorus run-off and subsequent eutrophication of aquatic ecosystems, as well as the release of greenhouse gasses, ozone layer degradation and acid rain, among other things [23]. PGPR can therefore be used for precision agriculture practices, which seek to optimise the usage of seeds, fertiliser and pesticides, while at the same time lowering costs and maximising yield quality and the economic return of a given agricultural production system [24,25]. Precision agriculture relies heavily on remote sensing applications to enable site-specific crop management. Such approaches are enabled by hyperspectral imaging, which can record images containing spectral data for a range of wavelengths in the light spectrum, typically in the visible and near-infrared (VNIR: 400–1000 nm) and shortwave infrared (SWIR: 1000–2500 nm) spectral regions [26]. Spectral information is spatially resolved and extended to parts of the light spectrum invisible to the human eye [27]. Hyperspectral imaging can be used to resolve different plant stresses that change the biochemical composition of leaf tissue, enabling non-destructive plant phenotyping [28]. Internal leaf structure and pigment composition vary depending on the stressor and can result in different spectral signatures, depending on biotic or abiotic stress [29], while chlorophyll and carotenoid pigment content and spatial distribution can be used to determine the presence of leaf diseases [30]. Previous research has also shown that hyperspectral imaging can be used to distinguish winter wheat (*Triticum aestivum* L.) growing in microbiologically richer soils [31] and to detect the beneficial effects of some PGPR, since PGPR-mediated higher uptake of nutrients to plants leads to improved leaf biochemistry [32].

The efficacy of *B. firmus* I-1582 as a bionematicide has been documented, and possible nematode virulence factors that could explain the nematicidal properties of this strain have been described within the assembly of the *B. firmus* I-1582 genome [12]. However, to the best of our knowledge, there are no studies examining the genetic capability of *B. firmus* I-1582 to promote plant-growth and concurrent nematicidal and plant-growth promoting effects on nematode-stressed tomato plants using hyperspectral imaging. The aims of this study were therefore (1) to investigate the nematicidal performance of *B. firmus* I-1582 against *M. luci* on tomatoes in a controlled environment in a glasshouse, as well as in a microplot experimental site serving as a field-analogue; (2) to evaluate the genetic capability of *B. firmus* I-1582 as a PGPR through bioinformatics analysis and compare it with the measured elemental composition of plant tissue; (3) to measure the effects of *B. firmus* I-1582 inoculation on plant physiology and substrate/soil biological activity; and (4) to test the ability of hyperspectral imaging to differentiate and characterise plants with different treatments. Our hypotheses were in line with the aims stated above: (1) *B. firmus* I-1582 has a marked nematicidal influence on *M. luci* on tomatoes; (2) as a PGPR, *B. firmus* I-1582 has the genetic capability to facilitate nutrient uptake in tomato plants; (3) *B. firmus* I-1582 has a positive effect on plant physiology and soil biological activity; and (4) hyperspectral imaging, combined with machine learning methods, can successfully distinguish between different treatments and identify relevant spectral regions for these differences.

## 2. Results

### 2.1. Nematode Reproduction and Plant Morphology

Nematodes reproduced very successfully in the pot experiment, as shown by the reproduction factor (R_f_) data for the non-treated plants in the negative control (NC) treatment. Treatment with a chemical nematicide—positive control (PC)—completely prevented *M. luci* infestation in pot experimental conditions, while biological control with *B. firmus* I-1582 (low *B. firmus* inoculum and high *B. firmus* inoculum treatments, BfL and BfH, respectively) effectively reduced nematode infestation levels by 46%–51%. High bacterial inoculum opposed to low bacterial inoculum did not produce any significant difference in nematode control (Table 1). In the microplot experiment, both chemical and biological nematicides significantly reduced the nematode infestation rate and did not differ significantly in efficacy. Inoculation with *B. firmus* did not significantly affect fresh root weight at the end of the microplot experiment, in contrast with the results from the pot experiment (Table 2).

**Figure 1:**
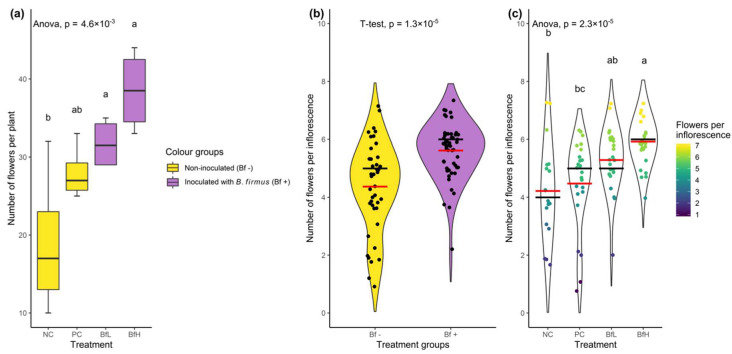
Data from the pot experiment showing the number of flowers per plant according to (**a**) treatment (dataset size, n = 16) and (**b**) the number of flowers per inflorescence in *Bacillus firmus*-inoculated/non-inoculated plants (n = 93), or (**c**) according to four tested treatments (n = 93). The numbers of flowers per plant are presented with the box plot and coloured according to the presence or absence of bacterial inoculum. Data for the number of flowers per inflorescence are presented with a violin plot with median (black bar) and mean values (red bar). The outline of the violin plot is a density function representing the underlying data frequency. Individual data points in the right-hand pane were coloured according to the number of flowers in each inflorescence (going from violet/blue representing smaller inflorescences to yellow for larger ones). Treatment means were separated using Tukey’s HSD (**a**,**c**) or a T-test (**b**). Means sharing a letter are not significantly different at *p* < 0.05. NC—negative control; PC—positive control (nematicide); BfL—low *B. firmus* inoculum; BfH—high *B. firmus* inoculum.

In the pot experiment, the total leaf area and associated plant dry weight results were negatively correlated with the nematode reproduction parameters. The leaf area was lowest in untreated plants (NC) and significantly higher in *B. firmus*-treated plants (BfL and BfH) (Table 3). The flower and inflorescence counts in each plant from the various treatments showed that the treated plants had a significantly higher number of flowers compared to the untreated plants (F_3, 12_ = 7.39, *p* = 0.0046). Additionally, *B. firmus*-inoculated plants had a higher number of flowers per inflorescence than the non-inoculated plants, with increasing instances of larger inflorescences per plant with a larger dose of *B. firmus* inoculum (Figure 1). The average fresh root weight increased with the addition of *B. firmus* inoculum, although this was statistically supported only for the BfH treatment. The mean height of the plants did not significantly differ in any of the treatments.

### 2.2. Photosynthetic Parameters

Measurements of photosynthesis (net photosynthetic rate and chlorophyll *a* fluorescence) were used to investigate the effects of different RKN control treatments, or their absence, on the plant’s physiological state. In the pot experiment, the effective quantum yield of photosystem II (PSII), apparent electron transport rate (ETR) and maximum quantum efficiency of PSII photochemistry (Fv/Fm) significantly differed between treatments, while the photosynthesis rate, stomatal conductance and transpiration did not (Table 4). The values for the photosynthesis rate, ETR and effective quantum yield of PSII in *B. firmus*-inoculated plants (BfL and BfH) were higher compared to non-inoculated plants (NC and PC); however, the differences were not statistically significant. Significance in the abovementioned parameters was observed between treated (PC, BfL and BfH) and untreated plants (NC). In the microplot experiment, the rate of photosynthesis, stomatal conductance, transpiration and ETR were not statistically different for the treatments, though the highest values were recorded for the BfH treatment. On the other hand, the effective quantum yield of the PSII and Fv/Fm values differed significantly in observations, with the lowest values for the NC treatment and the highest for BfH.

### 2.3. Presence of B. firmus I-1582 Bacteria and Microbial Activity of the Substrate

**Figure 2:**
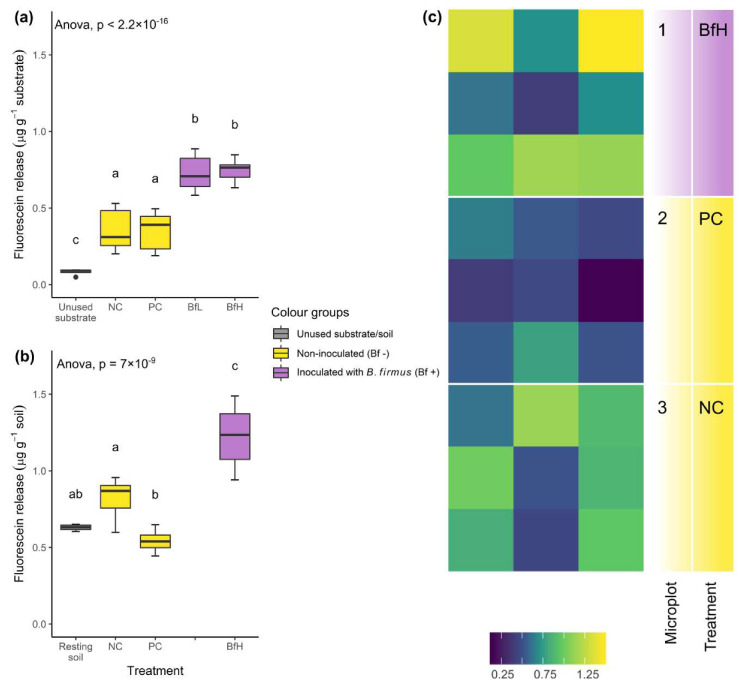
Microbial activity measurements of the substrate and soil from (**a**) pot experiments and (**b**) microplot experiments with different treatments. Data from the pot (n = 16) and microplot experiments (n = 12) are presented with a box-plot showing the results of ANOVA and Tukey’s HSD test. Treatments sharing a letter were not significantly different at *p* < 0.05 and are coloured according to the presence or absence of *Bacillus firmus* inoculum. (**c**) Spatially resolved total microbial activity in the microplot soil, as measured by fluorescein release (µg g^−1^), ranging from lower (violet-blue) to higher (yellow) enzymatic activity, presented in an area heatmap. NC—negative control; PC—positive control (nematicide); BfL—low *B. firmus* inoculum; BfH—high *B. firmus* inoculum.

The presence of viable *B. firmus* I-1582 in the rhizosphere was confirmed at the end of the pot experiment in the BfL- and BfH-treated plants, as well as in the BfH-treated plants in the microplot experiment. Inoculation of tomato plants with *B. firmus* in all instances significantly increased substrate/soil microbial activity compared to the non-inoculated plants (Figure 2). In the microplot experiment, the overall microbial activity of the soil was higher compared to the pot experiment, even in non-inoculated plants (Figure 2b). In microplots with non-inoculated plants (NC and PC), total microbial activity was non-specifically distributed across the microplot area, while in the microplot with *B. firmus*-inoculated plants (BfH), the fluorescein fluorescence significantly increased in the microplot corners closer to the plant roots (Figure 2c).

### 2.4. Bioinformatics Analysis, Pigment and Macro- and Microelement Composition of Tomato Leaves

**Figure 3:**
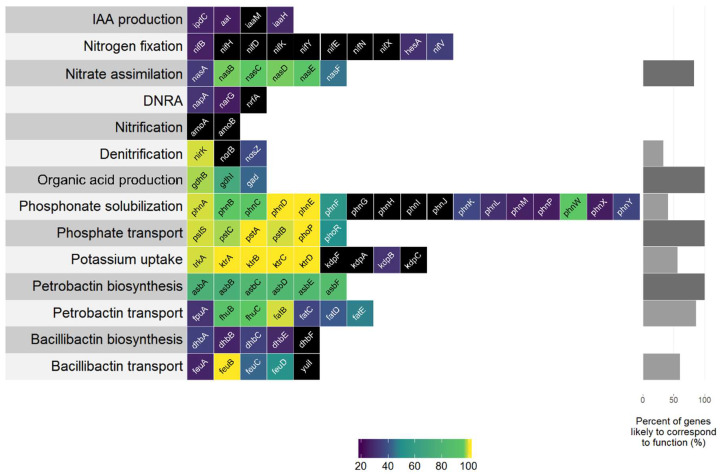
Gene homologs in the *B. firmus* I-1582 genome in various biochemical pathways and/or processes associated with the plant growth-promoting (PGP) effects of rhizobacteria. Each box represents a single gene queried, with boxes coloured according to the identity (%) for the query, ranging from a low (violet) to high identity (yellow). Black boxes indicate the absence of a gene within the I-1582 genome assembly. DNRA—dissimilatory nitrate reduction to ammonium.

A search for genes within the *B. firmus* I-1582 genome revealed the presence of various bacterial genes and biosynthetic pathways associated with plant growth promotion (PGP), while some biosynthetic pathways documented in rhizobacteria were lacking in *B. firmus* I-1582. The *nif* gene cluster encoding the capability for atmospheric nitrogen fixation was missing, as well as genes associated with phytohormone indole-3-acetic acid (IAA) production and siderophore bacillibactin biosynthesis. The strain was found to have genes associated with nitrogen assimilation, phosphate and phosphonate solubilisation and transport, potassium uptake as well as siderophore biosynthesis and transport (Figure 3), indicating the genetic capability of *B. firmus* I-1582 for PGP.

Relative chlorophyll content and elemental quantities in leaf tissue were analysed to evaluate differences between treatments in the microplot experiment. The relative chlorophyll content in leaves was significantly higher in *B. firmus*-treated plants and comparison of macro- and micronutrient quantities in leaves in plants (Table 5) showed significant differences between treatments for plants in terms of Ca, Cu, Mo, N, S and Zn content. *B. firmus*-treated plants had significantly lower quantities of Ca, S and Mo but a higher quantity of Zn. Although BfH plants also averaged higher N, K and Na content and lower quantities of Fe and Mn compared to plants in the NC and/or PC, these differences were not significant at *p* < 0.05. Further, permutational multivariate analysis of variance (npMANOVA) of nutrient abundance and distribution showed that *B. firmus* treatment had significant effects on the nutrient composition of plants in the microplot experiment (Table S4). Variability between groups was showcased by the principal component analysis (PCA), which also exhibited differences between samples in all three treatments, and samples clustered together into three groups according to treatments (Figure S3). Additionally, data for the different plant physiology, morphology, microbial activity and nematode reproduction variables in the pot and microplot experiment datasets were tested with npMANOVA and PCA. Both analyses revealed there were significant differences between treatments in the pot and microplot datasets at *p* < 0.05 (Tables S1–S3; Figures S1–S3).

### 2.5. Hyperspectral Imaging Analysis

**Figure 4:**
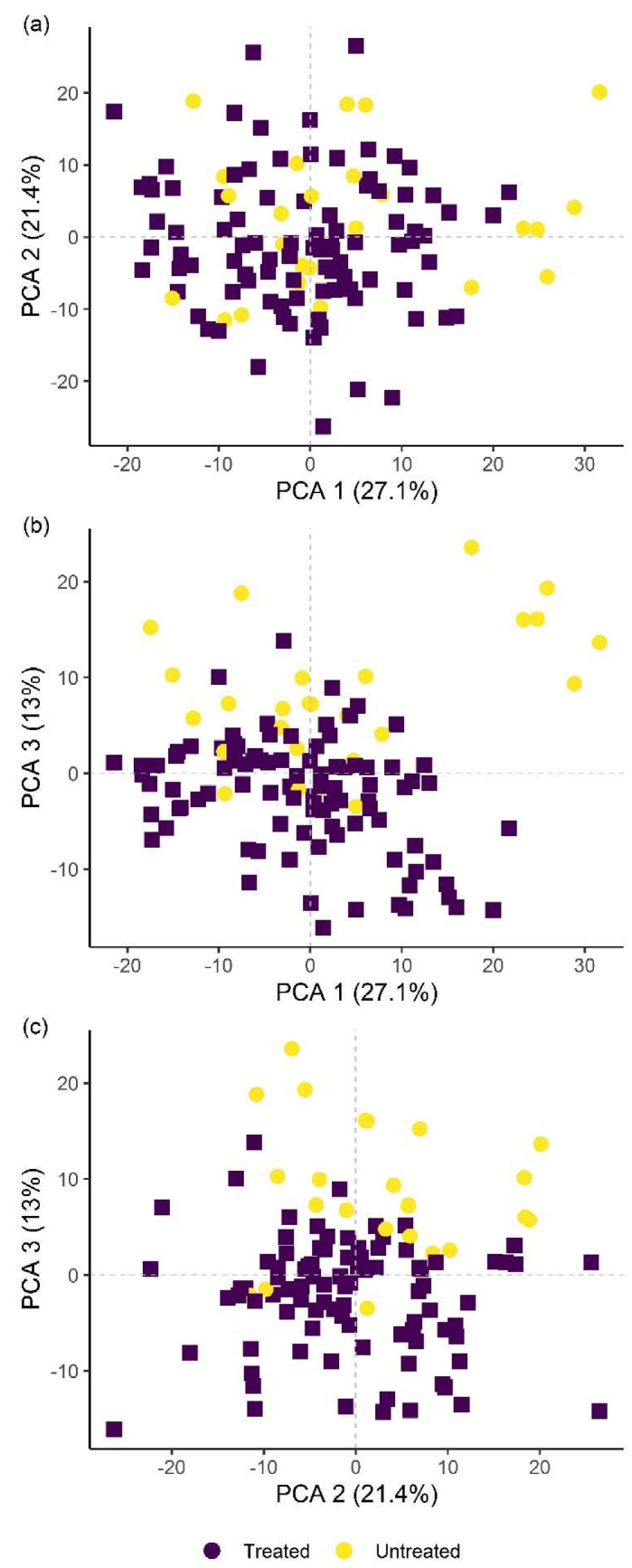
Principal component analysis (PCA) score plots showing the variation in plant groups from the pot experiment with regard to treated (PC, BfL and BfH) or untreated plants (NC). The first three principal components (PCA 1, 2 and 3) explain more than 60% of observed variability and are presented in the combinations (**a**) PCA 1–2; (**b**) PCA 1–3; and (**c**) PCA 2–3. NC—negative control; PC—positive control (nematicide); BfL—low *B. firmus* inoculum; BfH—high *B. firmus* inoculum.

**Figure 5:**
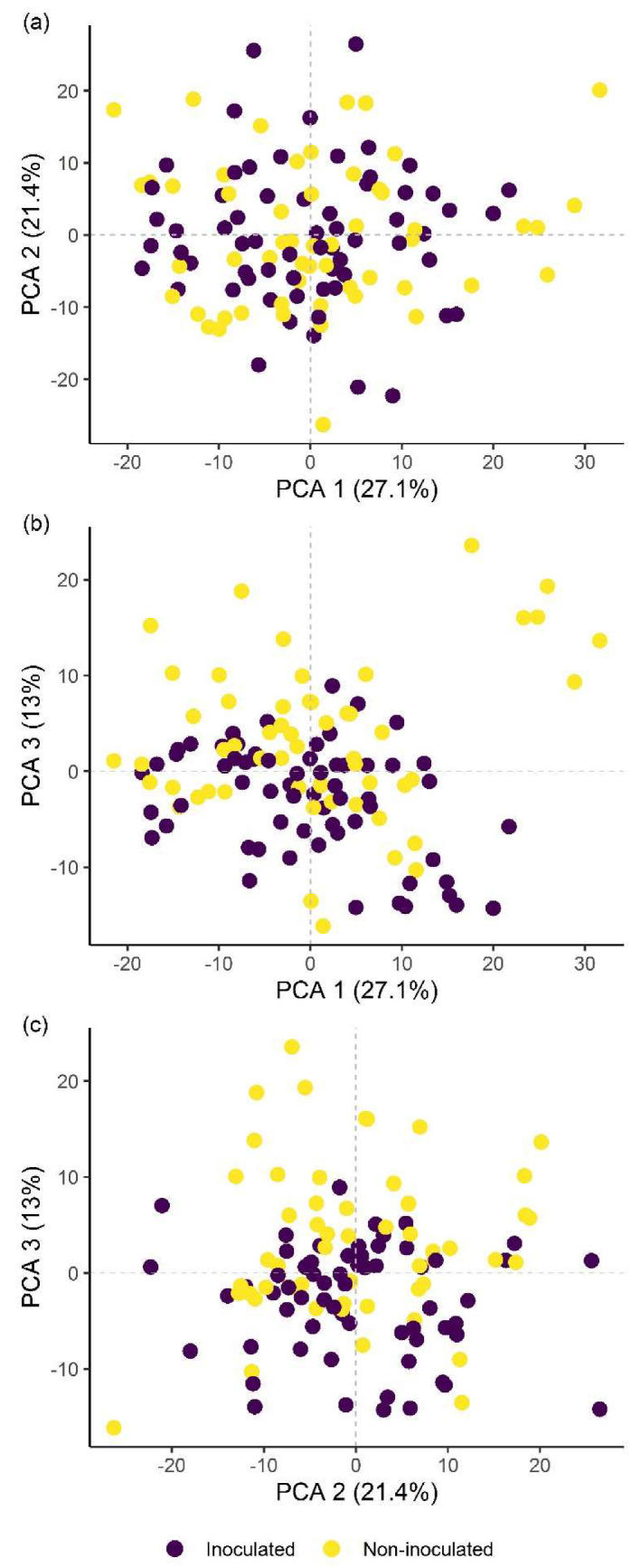
Principal component analysis (PCA) score plots showing the variation in plant groups from the pot experiment in *Bacillus firmus*-inoculated (BfL and BfH) or non-inoculated plants (NC and PC). The first three principal components (PCA 1, 2 and 3) explain more than 60% of the observed variability and are presented in the combinations (**a**) PCA 1–2; (**b**) PCA 1–3; and (**c**) PCA 2–3. NC—negative control; PC—positive control (nematicide); BfL—low *B. firmus* inoculum; BfH—high *B. firmus* inoculum.

**Figure 6:**
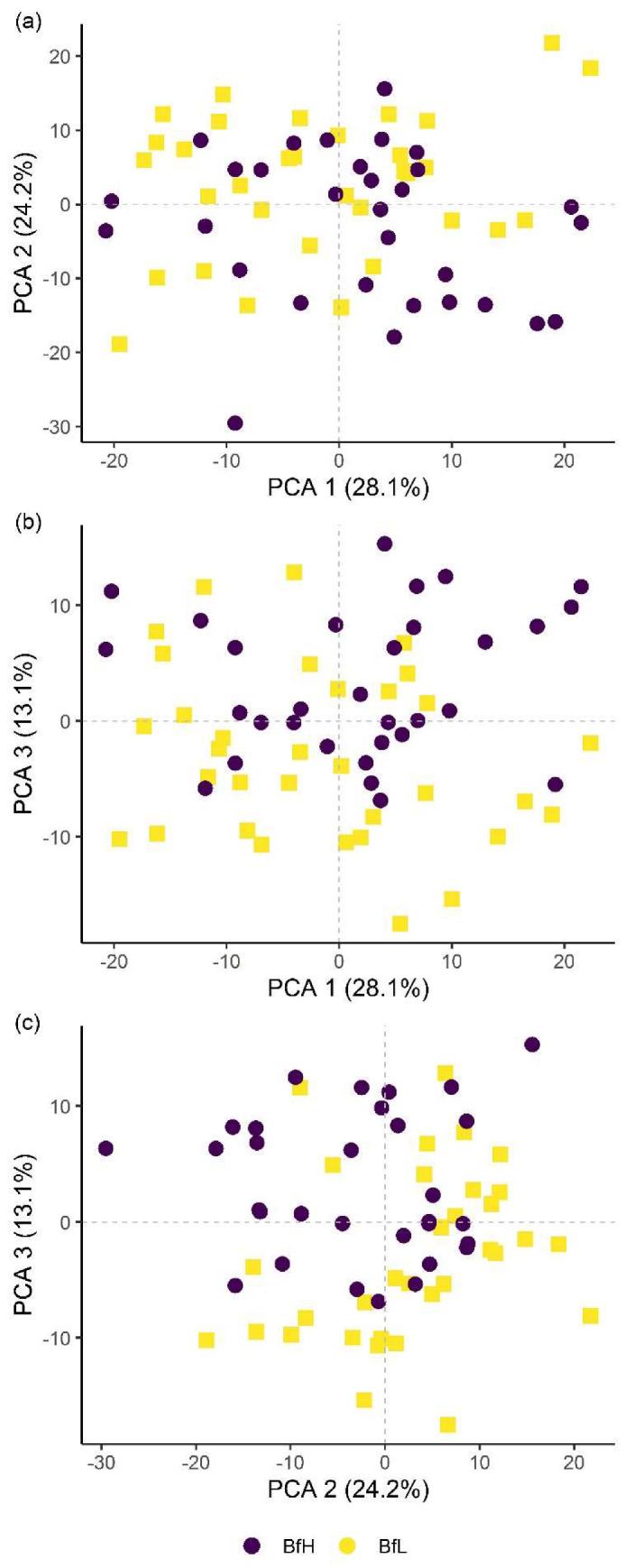
Principal component analysis (PCA) score plots showing the variation in plant groups from the pot experiment in plants treated with high (BfH) or low-*Bacillus firmus* inoculum (BfL). The first three principal components (PCA 1, 2 and 3) explain more than 65% of observed variability and are presented in the combinations (**a**) PCA 1–2; (**b**) PCA 1–3; and (**c**) PCA 2–3.

**Figure 7:**
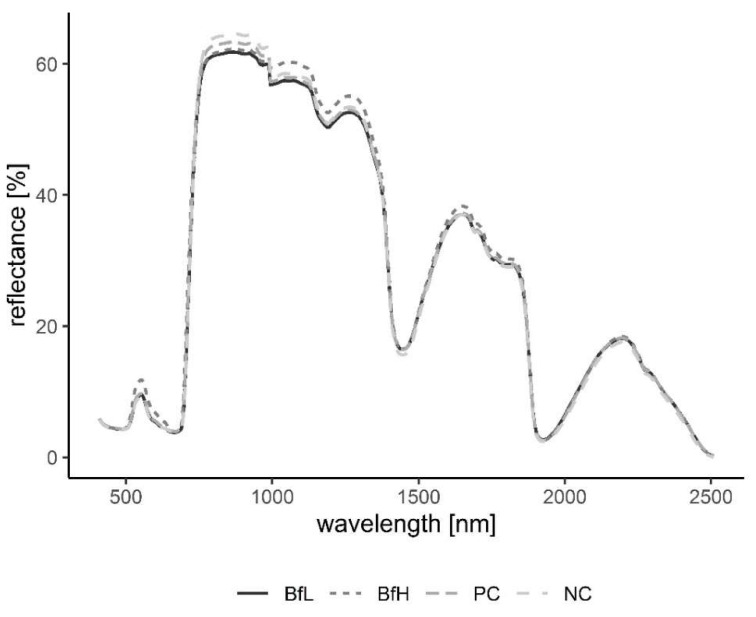
Averaged spectral signatures of the plants delineating the changes in reflectance at different wavelengths in the recorded spectrum. Signatures grouped according to treatments NC, PC, BfL and BfH in the pot experiment. Sample size per treatment, n = 4. NC—negative control; PC—positive control (nematicide); BfL—low *B. firmus* inoculum; BfH—high *B. firmus* inoculum.

**Figure 8:**
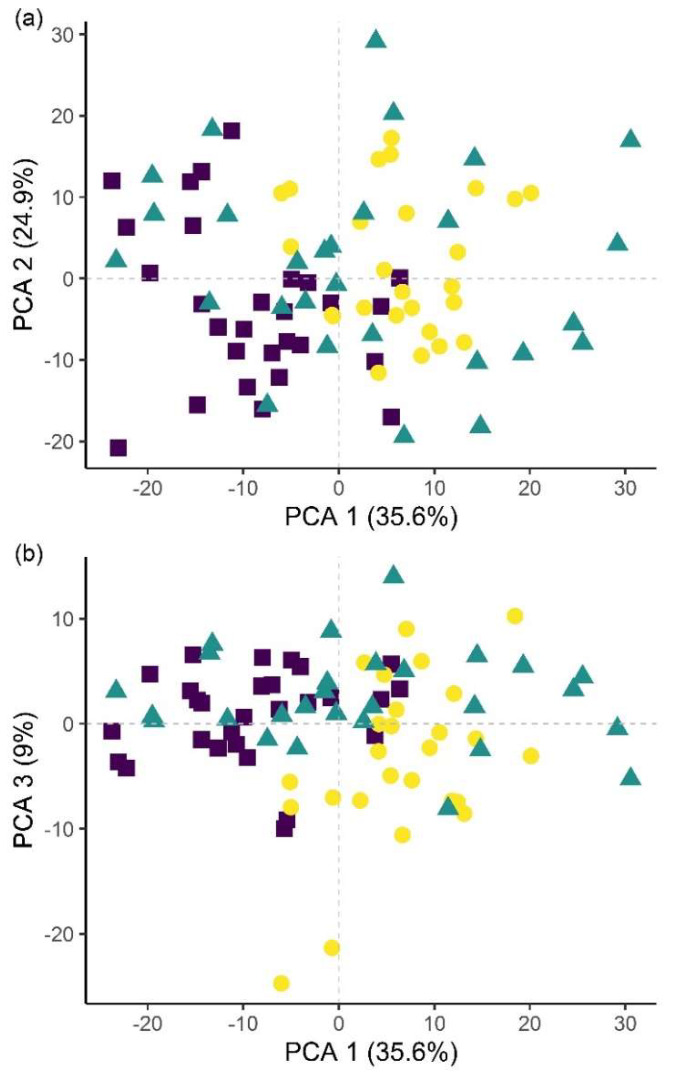

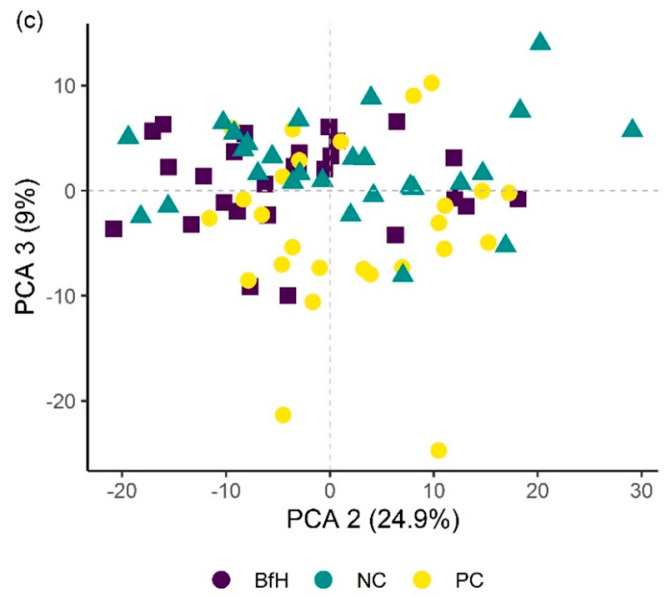
Principal component analysis (PCA) score plots showing the variation in plant groups from the microplot experiment in the *Bacillus firmus*-treated plants (BfH) and the negative (NC) and positive (PC) controls. The first three principal components (PCA 1, 2 and 3) explain more than 69% of observed variability and are presented in the combinations (**a**) PCA 1–2; (**b**) PCA 1–3; and (**c**) PCA 2–3.

**Figure 9:**
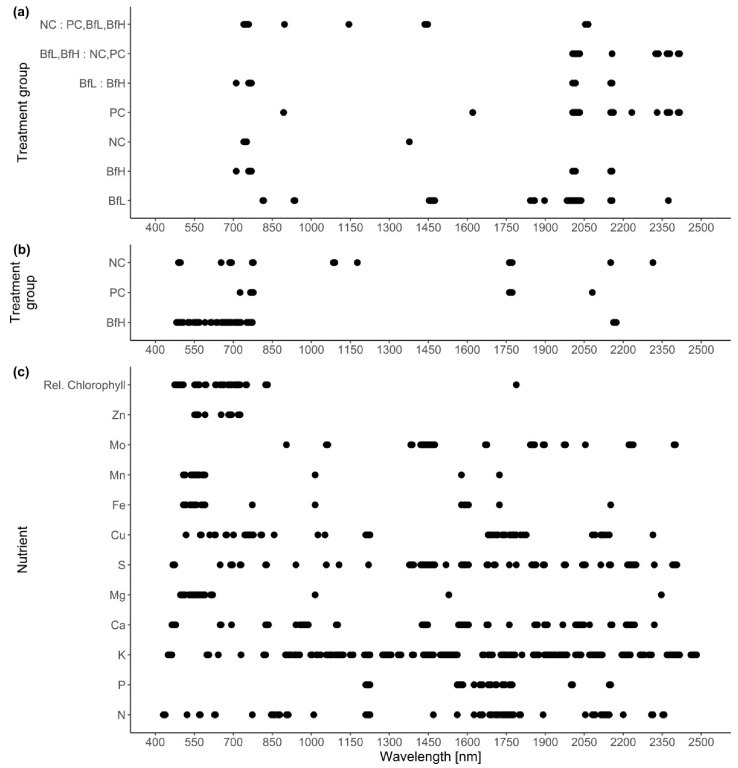
Loadings of the first partial least squares discriminant analysis (PLS-DA) (**a**,**b**) and partial least squares regression (PLS-R) (**c**) component for the pooled samples. The data points show spectral ranges with correlation values higher than 0.7 or lower than −0.7 in (**a**) the pot experiment, (**b**) the microplot experiment and (**c**) the nutrient content datasets. NC—negative control; PC—positive control (nematicide); BfL—low *B. firmus* inoculum; BfH—high *B. firmus* inoculum.

Classification of hyperspectral imaging data using partial least squares support vector machines (PLS-SVM) showed it was possible to discriminate between treated and untreated plants in both pot and microplot experiments. In the pot experiment, separation of the four treatment groups achieved an accuracy of 87.2% (Table 6). Further sample groupings were explored to improve the reliability of treatment identification. Accuracy of classification increased when samples were grouped into treated and untreated plants (100%), or *B. firmus*-inoculated and non-inoculated plants (97.4%), and also achieved 100% success when classifying different sizes of *B. firmus* inocula (BfL and BfH). These results are shown in the PCA score plots (Figure 4, Figure 5 and Figure 6), while changes in leaf reflectance at different wavelengths were further apparent in treatment-averaged spectral signatures (Figure 7). Separation of treatment groups using hyperspectral data was also successful in microplot experiment (Figure 8). Differences between treated (PC, BfL and BfH) and untreated (NC) plants in the pot experiment were characterised by reflectance in the ranges 740–759, 897, 1145, 1437–1448 and 2054–2065 nm. Discrimination between *B. firmus*-inoculated (BfL and BfH) and non-inoculated plants (NC and PC) occurred due to differences in the 2005–2033, 2157 and 2325–2417 nm ranges, while separation of the two inoculum sizes (BfL and BfH) was characterised by reflectance at 711, 759–770, 2005–2016 and 2152–2157 nm (Figure 9a). In the microplot experiment, separation of samples from all treatments (NC, PC and BfH) using PLS–SVM was more successful compared to the pot experiment, achieving an accuracy of 96.3% (Table 6). PCA analysis of the hyperspectral data separated the treatment groups, with the first three principal components explaining 69.5% of observed variance (Figure 8). Samples in the NC treatment were discriminated by reflectance in the ranges 489–496, 652, 686–693, 773–776, 1085–1090, 1177, 1762–1772, 2152 and 2314 nm, and in the PC treatment by reflectance in the 726–776, 1762–1773 and 2081 nm ranges, while samples from the BfH treatment showed significant reflectance across the whole visible range at 482–773 and SWIR at 2162–2173 nm (Figure 7 and Figure 9b). Spectral ranges that correlated with the contents of specific nutrients measured in leaf tissue from plants in the microplot experiment were determined through partial least squares regression (PLS-R) analysis (Figure 9c). For each nutrient PLS-R model, more than 80% variance was explained with the first three PLS components (Table S5).

## 3. Discussion

Inoculation of the tomato rhizosphere with *B. firmus* I-1582 had a dual effect on the plants. *B. firmus* reduced the severity of *M. luci* infestation in both pot and microplot experiments by approximately 50%, while at the same time having beneficial effects on plant morphology, physiology and nutrient uptake. *B. firmus*-treated plants exhibited PGP effects compared to the control plants (PC), where nematode infestation was completely eliminated (pot experiment) or inhibited to the same level (microplot experiment). The results indicated PGP effects of *B. firmus* were not associated solely with lower nematode counts, although the specific *B. firmus* PGP effects indicated by the results were not controlled using a separate treatment of *B. firmus*-treated plants not inoculated with nematodes. In the pot experiment, the plant physiology measurements only detected differences between treated (PC, BfL and BfH) and untreated plants (NC). Photosynthesis rate measurements did not show statistically significant differences between treatments despite seemingly clearly separated means. This was due to the high variance of the data in the negative control (NC) and a single aberrant measurement in the BfL treatment. Removal of this outlier resulted in statistically significant differences in photosynthesis rates for the treated (PC, BfL and BfH) and untreated (NC) plants (F_3, 11_ = 7.05, *p* = 0.0065). In the microplot experiment, a significant difference between treatments was only observed in the effective quantum yield of the PSII and Fv/Fm values, while the other parameters were not significantly different. In general, the highest rates of photosynthetic parameters were recorded for the BfH treatment. The small data sample, together with the high variability of the measured parameters recorded for the PC treatment, could explain the lack of significance for this treatment. Some morphological and biochemical parameters indicated plant growth promotion (PGP) of *B. firmus*, examples being leaf area, number of flowers, plant dry weight and fresh root weight in the pot experiment, as well as relative leaf chlorophyll content and the content of some elements in the microplot experiment. These PGP effects were evident even when compared to the positive control (PC), where the chemical nematicide was used to either completely eliminate the nematode infestation in the pot experiment (Table 1), or suppress it to the same level as in *B. firmus*-treated plants in the microplot experiment (Table 2), thus eliminating or levelling the effects of RKN-induced biological stress on tomatoes in the PC treatment compared to BfH. The efficacy of the chemical RKN control treatment (PC; active ingredient fluopyram) was inconsistent in the pot and microplot experiments. Lower nematode suppression in the PC in the microplot experiment could be attributed to experimental design and environmental factors. To approach field-like RKN infestation conditions in the microplot experiment, highly infested tomato roots were incorporated into the microplot soil. *M. luci* eggs were contained within the gelatinous matrix of the egg-masses on the root surface or even inside the roots, and were therefore shielded from the effects of chemical treatment to a greater extent than the eggs in suspension used in the pot experiment. A greater day/night temperature difference in the microplot experiment could also have resulted in more gradual J2 larvae hatching than in the controlled glasshouse environment. Thus, in the microplot experiment, a higher number of J2 larvae were invading plant roots between nematicide applications, when fluopyram was less efficient [33]. Additionally, the microplot soil was much more heterogeneous in terms of particle size and composition than the defined substrate used in the pot experiment, leading to possible areas of sub-optimal nematicide concentrations. Fluopyram has previously been shown to have a limited effect on *Heterodera glycines* inhibition beyond the applied area of the soil [34]. Although *B. firmus* consistently reduced *M. luci* infestation levels in the pot and microplot experiments (Table 1 and Table 2), previous studies have highlighted more variable results [35] or inconsistent success in controlling plant-parasitic nematode populations and crop yield [36,37]. However, the inconsistencies observed in these studies may be the result of variable environmental conditions, unsuccessful rhizosphere colonisation and microbial competition. Beeman and Tylka [37] further pointed out the need to evaluate the nematicidal action of *B. firmus* over longer experiments, which may also be important when characterising its PGP effects.

Inoculation with *B. firmus* I-1582 significantly increased the total microbial activity of the substrate or soil in both the pot and microplot experiments (Figure 2). Activity at the end of the experiment did not depend on the size of the bacterial inoculum, although the robustness of this observation was not tested in the microplots. Total microbial activity was lower in the pot experiment than in microplots, probably due to a higher sand content in the substrate used in the pot experiment. A high sand content can correlate with low fluorescein release, measured using the fluorescein diacetate (FDA) hydrolysis method [38]. The level of microbial activity in the BfH microplot treatment was similar to the results obtained by Giannakou et al. [14] when testing the same bacterial strain. Additionally, spatial analysis of FDA hydrolysis data in our experiment indicated the predominantly rhizosphere activity of *B. firmus* I-1582, since in the BfH microplot the highest fluorescence was measured in the areas closest to the plants, while the PC and NC microplots showed a more random distribution (Figure 2c).

Multiple genes associated with various PGP traits were found in the *B. firmus* I-1582 genome. Genes associated with phytohormone IAA production (*ipdC*, *aat* and *iaaH*) found in the I-1582 assembly did not show any similarity to query sequences that would be considered to correspond to function (Figure 3). Bacterially produced IAA can have PGP as well as deleterious effects on plant growth. IAA is associated with stimulating effects on root system architecture, increasing root hair formation and the number and length of the roots [39]. We did not observe significant differences in root system architecture in *B. firmus*-inoculated and non-inoculated plants in our experiments (results not shown). Fresh root weight was significantly higher in BfH plants in the pot experiment, but not in the BfH treatment in the microplot experiment (Table 2 and Table 3). The higher root weight in the BfH-treated plants in the pot experiment could be attributed to the larger plant canopy of these plants and not to the bacterial IAA-related effects on the root system.

With regard to nutrient uptake mechanisms, I-1582 was found to contain various gene homologues or metabolic pathways that could contribute to PGP (Figure 3). Nutrient measurements for leaves at the end of the microplot experiment were inconclusive in relation to the extent of actual PGP by I-1582 (Table 5). I-1582 did not appear to have the genetic capability for atmospheric nitrogen fixation, since the majority of genes from the *nif* cluster (*nifBHDKYENXhesAnifV*) did not have any matches in I-1582—especially the *nifH* gene, encoding the reaction-catalysing reductase subunit of nitrogenase [40]. Other nitrogen cycle-related genes without matches in I-1582 were *amoA* and *amoB* genes encoding the α- and β-subunits of the ammonia monooxygenase enzyme from *Nitrosomonas europaea* active in ammonia oxidation (NH_3_ to NO_2_^−^) as part of the nitrification process [41], and *norB* encoding the nitric oxide reductase enzyme involved in denitrification [42]. Homologues to the *nirK* and *nosZ* (weak identity) genes associated with denitrification were found in the I-1582 assembly. I-1582 also appeared to have the complete bacterial *nas* gene cluster (*nasABCDEF*) used for nitrate assimilation from dissolved and particulate organic nitrogen [43], but nitrate assimilated inside bacterial cells in such a manner would not be available to plants [41]. Sequences with weak identity to *napA* and *narG* involved in dissimilatory nitrate reduction to ammonium (DNRA) were found in I-1582. However, the gene *nrfA*, encoding the key nitrite reductase [44], was not found, indicating that I-1582 did not have the genetic capability for DNRA-associated PGP. Based on the bioinformatics analysis, this strain did not have the genetic capability for nitrogen fixation or DNRA, but rather used assimilated N in its own metabolism or facilitated its escape into the atmosphere through denitrification. On the other hand, various PGPR, such as *Bacillus* spp., have been demonstrated to enhance plant growth by influencing the expression of the nitrate (NO_3_^−^) and ammonium (NH_4_^+^) uptake genes of plants [45], which could potentially explain the higher average N content in BfH plants in the microplot experiment (Table 5). I-1582 was found to contain many gene homologues associated with mineral phosphate solubilisation through gluconic acid production (*gdh* and *gad*), phosphonate solubilisation (phosphonate gene cluster *phn*) and phosphate transport (*pst* operon: *pstSCAB* and *phoP*-*phoR* system). The phosphonate *phn* gene cluster enables the solubilisation of organophosphorus phosphonate compounds that are not otherwise bioavailable to plants. I-1582 did not contain all the genes from the clusters (Figure 3), similar to various *B. cereus* strains [46]. *B. firmus* is known to produce 2-ketogluconic acid and this organic acid secretion is considered the primary mechanism for phosphate, along with K and Zn solubilisation [20]. Various secondary mechanisms include the production of chelating substances and inorganic acids. Soil bacteria usually uptake solubilised phosphate and K through various types of transporters, such as Trk, Kdp and Ktr [47]. Homologues to genes from *ktrABCD*, but not to the *kdpFABC* operon, were found in the I-1582 genome. Nutrient measurements showed that although the BfH-treated plants averaged the highest K content in leaves, there were no significant differences between treatments, and all plants had borderline-deficient K concentrations [48]. No significant differences in P content were observed between treatments either, but the BfH-treated plants had a significantly higher Zn content (Table 5). Additionally, genes homologous to the siderophore petrobactin (anthrachelin) biosynthetic cluster (*asbABCDEF*), but not the bacillibactin (*dhbABCEF*) biosynthetic cluster, were found in the I-1582 genome. Siderophores are low molecular weight Fe^3+^ chelators expressed into an extracellular medium, and petrobactin and bacillibactin are known to be produced by various *Bacillus* spp. under Fe-limited conditions [49,50]. Siderophores are considered PGP factors since solubilisation of Fe^3+^ increases Fe bioavailability to plants as well as microorganisms [20]. The presence of homologues to petrobactin biosynthesis and transport genes within the genome (Figure 3) indicated the genetic capability of I-1582 for some kind of siderophore production and possible PGP through enhancement of Fe uptake, but no significant differences were observed between treatments. In the microplot experiment, the BfH-treated plants had a significantly lower Ca content and higher (not statistically significant) average content of Na compared to the NC and PC. In low K^+^ conditions plants can uptake more Na^+^, which negatively affects the assimilation of Ca^2+^ [51], so the observed results were probably due to the plant’s physiological response and were not influenced by *B. firmus*, since PGPR are more likely to increase ion homeostasis, such as the K^+^/Na^+^ and Ca^2+^/Na^+^ ratios [52].

Plants in the pot experiment showed a typical spectral curve for green plants, exhibiting a well-defined reflectance peak around 550 nm (green band) and absorbance at 650–700 nm (red) and 400–500 nm (blue) by chlorophyll (Figure 7). The red-edge spectral region (690–750 nm) was characterised by a sudden increase in reflectance, indicating normally functioning plants. Reflectance curves in the NIR (approximately 750–1300 nm) and SWIR (1300–2500 nm) regions were also typical, with weaker water absorption bands at 980 and 1150 nm and well-defined water absorption features at 1450 and 1950 nm [53]. The differences in spectral signatures for the treatments were subtle and most apparent in BfH plants. In contrast to the observed morphology (Table 3, Figure 1), the reflectance spectra of the BfH pot experiment plants indicated stress responses. The BfH treatment resulted in higher reflectance in the green band, indicating lower chlorophyll content, while reflectance levelled with the BfL plants in the red-edge and NIR up until about 980 nm, where it again deviated from other treatments in the SWIR region. Higher average reflectance at 550–680 nm has previously been observed with various forms of stress, such as heavy-metal toxicity [54] or bacterial infestation of leaf tissue, leading to a lower chlorophyll content [30]. Interestingly, this effect was only observed in BfH-treated plants, but not in plants treated with low *B. firmus*-inoculum (BfL). As there were no significant differences in nematicidal effects between the BfL and BfH treatments (Table 1), the observed responses of the BfL plants could be attributed to induced systemic stress tolerance elicited by low inoculum of *B. firmus*, while high bacterial loads in the rhizosphere are known to occasionally have phytotoxic effects [16]. Furthermore, PGPR can induce a jasmonic acid (JA)-dependent signalling pathway, as seen with *Serattia marcescens* and *Bacillus pumilus*, leading to systemic stress resistance in *Arabidopsis thaliana* against viruses [55]. JA was shown to augment resistance to a broad range of herbivores, including RKN [56], and also increase the pigment content in leaves of *Nicotiana tabacum* [57]. On the other hand, the apparently lower chlorophyll content in BfH, as indicated by reflectance, could simply occur due to structural changes in leaf mesophyll, as shown by the spectral signatures in NIR (700–1300 nm). Gitelson et al. [58] observed that an increase in leaf thickness and/or density led to an increase in reflectance in NIR and to a per volume decrease in chlorophyll concentration (thus causing an increase in reflectance in the green band). Relative chlorophyll content measurements, however, do not appear to be affected by leaf thickness in this way [59]. The BfH treatment also led to higher reflectance in parts of the NIR and SWIR spectrum, which has previously been observed in plants subject to salt [60] or water stress [29,61,62]. This observation again contradicted the morphological data, since the BfH plants had larger canopies and a greater plant dry weight and fresh root weight—not indicative of persistent water stress. The spectral data could be explained by brief periodic water-limiting conditions due to high plant growth and limiting substrate volume towards the end of the pot experiment, especially since the discrepancies described above were not observed in the BfH-treated plants in the microplot experiment. Apart from foliar water content, however, the significant spectral ranges identified for discrimination of the treatment groups in both the pot and microplot experiments underlined the importance of dry matter-associated spectral regions (1.7, 2.1 and 2.3 μm) in the SWIR [63]. Wavelengths in the ranges 740–759 nm, linked to biomass reflectance, 1437–1448 nm, linked to water absorption [64], and 1145 nm and 2054–2065 nm, linked to the N–H stretch in proteins [65], were important in differentiating between the treated and untreated plants in the pot experiment; in turn, discrimination between *B. firmus*-inoculated and non-inoculated plants was determined by differences in the ranges 2005–2033 nm, linked to foliar water, lignin or cellulose, 2157 nm, linked to the C–N and N–H bonds in proteins, and 2325–2417 nm, linked to cellulose, starch, amylose and proteins. Apart from spectral ranges linked to biomass (759–770 nm) and water absorption (2005–2016 nm), the C–N and N–H stretches (2152–2157 nm) were also significant for separation of the two *B. firmus* inoculum sizes (Figure 9a). Nitrogen, probably contained in the molecular structures of the proteins, energy storage metabolites and structural molecules, were thus implicated as significant factors in discrimination between *B. firmus*-inoculated and non-inoculated plants. Significant spectral ranges in the BfH-treated plants in the microplot experiment were especially linked to chlorophyll (Figure 9c) and pigment content (482–773 nm), which was supported by relative chlorophyll measurements, as well as to proteins (2162–2173 nm). It has been reported that the relationship between relative chlorophyll content and leaf N content per leaf area is affected by environmental factors and the leaf features of crop species [66]. The spectral ranges linked to the C–N and N–H stretches of the proteins were equally important in the NC plants (1085–1090, 1177, 2152 nm; Figure 9b), which corresponded with the nutrient measurement data, since the BfH and NC plants in the microplot did not significantly differ in total leaf N content (Table 5, Figure S3). Differences between the treatments described above made it possible to differentiate between *B. firmus*-treated and untreated plants using hyperspectral remote sensing, with a reliability of 96.3% (microplot experiment) and 97.4% (pot experiment). This analysis showcased the possibility of using hyperspectral remote sensing and image analysis to determine successful application of nematicidal and plant-growth-promoting bacterial agents in the rhizosphere. Successful application of a biological control agent means successful growth of added bacteria in the rhizosphere, through which they produce metabolites having nematicidal and PGP effects. Detailed plant-by-plant determination of a successful biological control agent application could be determined using hyperspectral remote sensing and image analysis, and a secondary application of bacteria could be prescribed for specific plants not yet protected by the first application.

## 4. Materials and Methods

### 4.1. Experimental Design and Plant Preparation

Experiments were designed to test the capability of *B. firmus* I-1582 (VOTiVO^®^, Bayer Crop Science, Monheim am Rhein, Germany) to control RKN *M. luci* infestation in the controlled environment of the glasshouse and in field-analogous microplots. The full genome sequences of both the pest *M. luci* and the biological control agent *B. firmus* I-1582 have been recently determined [12,67]. Nematode-infested plants were subjected to four treatments with four biological replicates per treatment as follows: untreated plants (NC, negative control); plants treated with the chemical nematicide Velum^®^ Prime SC (Fluopyram (Pyridinylethylbenzamide), Bayer Crop Science) by drenching the root zone with a 0.625 L/ha concentration, according to the manufacturer’s instructions (PC, positive control); plants treated with the VOTiVO product (BfL, Low *B. firmus* inoculum) according to the manufacturer’s instructions, in the form of a seed treatment; and VOTiVO seed treatment followed by additional drenching with VOTiVO suspension (BfH, High *B. firmus* inoculum) after transplanting (Table 7). The commercially available tomato (*S. lycopersicum*) hybrid “Horus F1” (L’Ortolano, Forlì-Cesena, Italy) was used in the experiments. Seeds were first surface sterilised in 3% aqueous solution of sodium hypochlorite (NaOCl; Kemika, Zagreb, Croatia) and afterwards coated either with 2% (w/v) aqueous solution of carboxymethyl cellulose (CMC; Sigma-Aldrich, Steinheim, Germany) to be used in the NC and PC treatments, or with CMC and VOTiVO suspension containing 2 × 10^6^ bacterial spores per seed, to be used in the BfL and BfH treatments. CMC was added to enable spore adhesion to the seed surface, as described by Razinger et al. [68]. Coated tomato seeds were potted in sterile 10 cm-diameter polypropylene pots (V = 0.5 L) with autoclaved substrate and allowed to germinate and grow for 49 days. Plants were grown on separate trays depending on whether they were inoculated (Bf+) or non-inoculated (Bf−) with *B. firmus* I-1582 (Table 7).

### 4.2. Pot Experiment

The experiment was conducted in a glasshouse at the Agricultural Institute of Slovenia (Ljubljana, Slovenia) from April to July 2019. A total of 49-day-old plants were transplanted to 13 cm-diameter pots (V = 1 L) and supported with 1 m plastic-coated stakes. Four treatments—NC, PC, BfL and BfH—were evaluated in the pot experiment (Table 7). Tomato plants were inoculated with 4 × 10^3^ *M. luci* eggs (representing 4 eggs/cm^3^ of substrate). The following day, plants in the PC treatment were watered with chemical nematicide solution, and plants in the BfH treatment were watered with a VOTiVO suspension containing additional 2 × 10^10^ *B. firmus* I-1582 spores per plant. Each pot was equipped with a saucer, and plants from the same treatment were contained within the same tray to prevent cross-contamination during watering. The growing conditions in the greenhouse chambers were as follows: 22 °C (±2 °C), 60%–70% air humidity, with natural daylight. The pot experiment was terminated 48 days after nematode infestation (DAI) when *M. luci* was expected to complete its first developmental cycle. The total number of nematode eggs per plant, eggs per gram of roots, the resulting nematode reproduction factor (R_f_) and galling index (using the scale based on Zeck et al. [69], ranging from 0—no infestation, healthy plant and roots to 10—heavy infestation, plant and roots dead) were calculated. Procedures for substrate preparation, fertiliser type, RKN egg preparation and counting, as well as for calculation of R_f_, were described previously in Susič et al. [29].

### 4.3. Microplot Experiment

The experiment was conducted at the microplot installation in the Agricultural Institute of Slovenia (GPS coordinates: 46.061402, 14.519247) from April to September 2019. Concrete-barrier microplots (1 m^2^) enabled the contained (quarantined) study of soil pest control treatments under field-like conditions. The soil within the microplots was determined to be of the sandy loam type, consisting of 48.2% coarse sand, 23% fine sand, 20.9% silt, 7.8% clay and 4.4% organic matter (pH = 7.4). Prior to planting, the soil was adjusted with Potgrond H (80 L/m^2^) peat substrate (Klasmann-Deilmann, Geeste, Germany), and finely cut tomato roots heavily infested with *M. luci* (egg-masses visible on the root surface) were incorporated in the soil to a final concentration of 250 × 10^3^ eggs/plant. Three treatments—NC, PC and BfH—were evaluated in the microplot experiment (Table 7). The microplot in the PC treatment was watered with the chemical nematicide Velum Prime, and the microplot in the BfH treatment was watered with a VOTiVO suspension containing 8 × 10^10^/m^2^ *B. firmus* I-1582 spores. Two days later, the 49-day-old tomato plants were transplanted in microplots (four plants per microplot). The microplots were fertilised once at 14 DAI with 50 g/m^2^ of NPK 15–15–15 fertiliser (Unichem, Sinja Gorica, Slovenia). Preventive management of late blight (*Phytophthora infestans*) on tomatoes was achieved using the fungicide Acrobat^®^ MZ (Dimethomorph + Mancozeb, BASF), which was applied twice according to the manufacturer’s instructions, on the 27th and 52nd day after the plants were transplanted. Plants in all treatments were sprayed equally. The environmental conditions from April to September 2019 were as follows: mean monthly temperature 18.2 °C (ranging from 11.6 to 23.3 °C), mean monthly precipitation 106.6 mm (ranging from 7.2 to 236.2 mm) and mean monthly solar irradiation of 131.1 kW/m^2^ (ranging from 95.8 to 179.7 kW/m^2^). The microplot experiment was concluded at the end of the growing season at 114 DAI. To evaluate nematode infestation and survival, plant roots were examined and assigned galling indices, while the soil in each microplot was sampled with a soil probe to a depth of 20 cm. Nematodes were extracted using a modified Baermann funnel method [70] and counted under a stereomicroscope Nikon SMZ800 (Nikon, Tokyo, Japan).

### 4.4. Plant Photosynthesis and Morphology

Plant photosynthetic parameters and morphology were recorded at 48 DAI in the pot experiment, and at 114 DAI in the microplot experiment. Gas exchange and chlorophyll *a* fluorescence measurements were performed on the third to fifth fully expanded leaf from the apical shoot [71]. Photosynthesis rate, transpiration rate, stomatal conductance and apparent electron transport rate (ETR) were measured on one leaf per plant with the LI-6400XT Portable Photosynthesis System (LI-COR Biosciences, Lincoln, NE, USA). In the pot experiment, the measurements were taken between 10:15 and 11:50 at the following operating parameters: ambient air temperature 22.1 °C–27.3 °C, air humidity 44.3%–59.4%, reference CO_2_ concentration 380 μmol mol^−1^ and stable light intensity of 1000 μmol photons m^−2^ s^−1^ (internal LED source). The measurements in the microplot experiment were taken between 13:30 and 14:30 at the following operating parameters: air temperature 22.1 °C–27.3 °C, air humidity 45.3%–53.6%, reference CO_2_ 380 μmol mol^−1^ and stable light intensity of 1000 μmol photons m^−2^ s^−1^. Chlorophyll *a* fluorescence parameters were measured with a Mini-PAM pulse-amplitude-modulated fluorometer (Heinz Walz GmbH, Effeltrich, Germany). Parameter maximum quantum efficiency of photosystem II (PSII) photochemistry (Fv/Fm) was measured on 10-min dark-adapted leaves (one leaf per plant), while the effective quantum yield of PSII (Yield) was measured on light-adapted leaves (five data points on two leaves per plant). Due to the design and duration of the microplot experiment, the plant morphology data (number of flowers per inflorescence, total leaf area, plant height measured at the tip of the apical shoot and plant dry weight) were only recorded in the pot experiment. Total leaf area was measured using an LI-3100C Area Meter (LI-COR Biosciences) and plant dry weight was measured after drying at 55 °C for 4 days. Root fresh weight was recorded in both experiments.

### 4.5. Hyperspectral Imaging and Analysis

Hyperspectral imaging was conducted at 48 DAI in the pot experiment and at 113 DAI in the microplot experiment. The selected leaves used for physiology measurements were cut from the plants and placed on the laboratory rack set up for automated hyperspectral imaging (Norsk Elektro Optikk AS, Skedsmokorset, Norway) so that data acquisition across the spectrum was completed in 4 min per sample. Selected leaves from plants in the microplot experiment were collected, placed on ice and imaged within 1 h. The imaging arrangement reduced the complexity of downstream image pre-processing and analysis due to homogenous illumination, simplified sample geometry and eliminated specular light reflections from surrounding surfaces. The two pushbroom imaging spectrometers; HySpex VNIR (spectral range 400–988 nm) and SWIR (950–2500 nm) (Norsk Elektro Optikk AS) were mounted perpendicularly above the sample, placed on the linearly moving stage at a 30 cm lens-to-sample distance. The speed was synchronised with the scanning cameras frame rate and illumination intensity and was controlled by the data acquisition unit, installed according to the manufacturer’s instructions. The sample was illuminated by two calibrated halogen lamps with homogenous light intensity between 400 and 2500 nm, mounted next to the cameras to optimally illuminate the scan area below each camera. A calibrated diffuse white reference plate with 20% reflectance (SphereOptics, Herrsching, Germany) was included in each image. In order to increase signal to noise ratios, each scanning line was recorded three times and the results averaged. Reflectance for each band of every image pixel (R), image pre-processing and analysis were performed as described previously [72].

### 4.6. Presence of Inoculated Bacteria and Microbial Activity of the Substrate

In order to test for the presence and survival of the inoculated bacterial strain, rhizosphere samples were collected and sporogenic bacteria were isolated as adapted from Földes et al. [73] and Agrawal et al. [74] at the end of the experiments. *B. firmus* I-1582 colonies were identified using real-time PCR [75]. The fluorescein diacetate (FDA) hydrolysis method described by Adam and Duncan [38] was used to measure the effect of added microbial inoculum on total microbial activity of the substrate compared to non-inoculated controls. Unused, autoclaved substrate was included to measure the native hydrolytic activity of organic substrate matter in the pot experiment. In the microplot experiment, the 1 m^2^ total area of each microplot was divided into a 3 × 3 square grid and the soil from each of the nine parts was sampled once in the centre at a depth of 10 cm and tested with the FDA method to obtain spatially resolved microbial activity data for the microplot. Native soil activity in the microplot experiment was measured in the soil sample from the resting microplot not used in any experiments that year.

### 4.7. Bioinformatics Analysis

The *B. firmus* I-1582 genome assembly [12] was searched for the presence of genes associated with the plant growth-promoting effects of rhizosphere bacteria selected from the literature. Query gene amino-acid sequences were procured from the UniProt database [76] and searched against the I-1582 assembly using blastp, with an E-value threshold at 10^−5^.

### 4.8. Relative Chlorophyll Content and Macro- and Micronutrient Measurement

Relative chlorophyll content was measured with a MultispeQ v2.0 chlorophyll meter using the protocol recommended by the manufacturer (PhotosynQ, East Lansing, MI, USA) in selected leaves from microplot experiment plants previously used for physiology and hyperspectral measurements. The leaves were frozen at −20 °C to determine macro- and microelement composition. Frozen samples were freeze-dried using two 24 h cycles in a freeze dryer (Gamma 1-20 LMC-2, Christ, Osterode am Harz, Germany) and homogenised for 3 min at 30 Hz in a mixer mill (MM 400, Retsch, Haan, Germany) using zirconium oxide-lined balls and grinding jars. Homogenised samples were dissolved using microwave-assisted nitric acid (HNO_3_) digestion, prior to analysis with inductively coupled plasma mass spectrometry (ICP-MS) [77]. Additionally, total nitrogen content was determined using the Kjeldahl method [78].

### 4.9. Statistical Analysis

The data was checked for assumptions of homoscedasticity using Levene’s test. Data were then statistically analysed with ANOVA, and when statistically significant, with Tukey’s HSD (honest significant difference) test at α = 0.05 to separate means. Due to unequal variance between groups, the data for stomatal conductance and Fv/Fm from the pot experiment and yield from the microplot experiment were analysed with Welch’s ANOVA and the Games–Howell test. Data for the galling index and fluorescein release in the microplot experiment were transformed using the Box–Cox method to achieve normality. Statistical analysis was performed with R [79]. Data were presented as means with standard deviation (n ± SD) followed by statistical analysis results. Permutational (non-parametric) multivariate analysis of variance (npMANOVA) was conducted in R using the “adonis” function in the “vegan” package (v2.5-6) [80] and principal component analysis (PCA) with the R package “ggbiplot” (v0.55) [81] on the pot and microplot experiment variables, which were min–max normalised to the 0–1 range. Statistical evaluation of hyperspectral data followed the workflow from Žibrat et al. [72]. Leaf area pixels were extracted from individual leaflets and the reflectance data were smoothed using a Savitzky–Golay filter with second-order polynomials. In order to emphasize the small spectral variations and remove scattering effects, second-order derivatives were calculated. Data was split into train and test (validation) sets, containing 67% or 33% of instances, respectively. In the pot experiment the train set thus contained reflectance data of 80 leaflets, and test set of 39 leaflets. In the comparison between high and low *B. firmus* inocula (BfH and BfL, respectively), this distribution was 43:21 leaflets per set. In the microplot experiment the train set contained data from 55 leaflets, and the test set from 27 leaflets. Partial least squares regression (PLS-R) and discriminant analysis (PLS-DA) were used to reduce data dimensionality and identify the relevant spectral regions. PLS-DA scores were used as variables in non-linear support vector machine classification (PLS-SVM), using the radial basis function kernel. For each PLS-SVM classification the best combination (i.e., yielding the highest classification accuracy) of capacity factor (C) and gamma value were determined by performing a grid search on a log scale. Image data pre-processing and processing were performed in Envi 5.1 (Harris Geospatial, Broomfield, CO, USA), R and Unscrambler 10.3 (CAMO Software, Oslo, Norway). Graphic data representation was carried out with RStudio [82] using the “ggplot2” [83] and “Superheat” [84] packages.

## 5. Conclusions

This study demonstrated the comparable and substantial nematicidal activity of *B. firmus* I-1582 in pot and microplot experiments, as well as the usefulness of hyperspectral imaging for evaluating the effects of I-1582 inoculation in the tomato rhizosphere with concurrent *M. luci* infestation. I-1582 was shown to have multimodal nematicidal and PGP effects, evident in the inhibition of nematode reproduction and beneficial effects on plant morphology and, to some extent, nutrient composition. The specific mechanism behind the latter could not be determined in this study, but I-1582 was shown to have the genetic capacity for various nutrient uptake mechanisms. In addition, I-1582 might alternatively induce the plant’s own systemic response pathways, leading to the PGP effects observed. The effects of *B. firmus* treatments were shown, with reliable differentiation from non-inoculated plants in hyperspectral image analysis. Spectral signatures linked to N-containing bonds of proteins, energy storage metabolites and structural molecules were significant for discrimination between *B. firmus*-inoculated and non-inoculated plants in the pot experiment, while in the microplot experiment the spectral ranges linked to chlorophyll, pigment content and proteins were significant. As a remote sensing approach, hyperspectral imaging could be an important tool for monitoring treatment success in the future, especially due to the documented variable performance of biological control agents such as *B. firmus*. Hyperspectral imaging also enabled a rapid assessment of the concurrent nematicidal and PGP effects of *B. firmus*, but future detailed studies into the biochemical basis of plant–microbe–nematode interactions would be needed to fully understand the observed multimodal effects of *B. firmus*. Hyperspectral remote sensing nevertheless enables sustainable precision agriculture by providing spatially accurate information prior to the development of visible symptoms of abiotic or biotic stress. It thus facilitates timely and accurate crop management, thereby reducing crop losses and management costs. Furthermore, remote sensing methods also enable monitoring of the survival and performance of biological control agents. Such monitoring could be useful due to the complexity of rhizosphere environments.

## Figures and Tables

**Table 1:** Evaluation of *Meloidogyne luci* infestation in tomatoes in the pot experiment.

Treatment	Galling Index (0–10)	Number of Eggs/Plant (*n* × 10^5^)	Number of Eggs/g Roots (*n* × 10^4^)	Reproduction Factor (R_f_)
NC	4.6 ± 0.3 a	7.4 ± 0.3 a	1.8 ± 0.2 a	185.2 ± 7.0 a
PC	0 ± 0.0 b	0 ± 0.0 b	0 ± 0.0 b	0 ± 0.0 b
BfL	3.6 ± 0.5 c	3.6 ± 1.3 c	0.8 ± 0.3 c	89.8 ± 32.1 c
BfH	3.3 ± 0.3 c	4.0 ± 0.8 c	0.7 ± 0.2 c	99.7 ± 20.8 c
ANOVA statistics	n = 16, F_3, 12_ = 171.11, ***p*** < **0.0001**	n = 16, F_3, 12_ = 61.57, ***p*** < **0.0001**	n = 16, F_3, 12_ = 58.72, ***p*** < **0.0001**	n = 16, F_3, 12_ = 61.57, ***p*** < **0.0001**

Root galling index scale (0—no infestation, 10—completely infested). Data presented as averages with the standard deviation (±SD); ANOVA statistic (n—dataset size, F_df, df_—F-statistic with degrees of freedom (df) between groups, and df within groups; *p*—*p*-value); and Tukey’s HSD results. Means sharing a letter are not significantly different at *p* < 0.05. NC—negative control; PC—positive control (nematicide); BfL—low *B. firmus* inoculum; BfH—high *B. firmus* inoculum.

**Table 2:** Evaluation of *Meloidogyne luci* infestation in tomatoes in the microplot experiment.

Treatment	Galling Index (0–10)	Number of Nematodes (g^−1^ Soil)	Root Fresh Weight (g)
NC	7.8 ± 0.5 a	2.9 ± 0.4 a	130.8 ± 30.7
PC	4.8 ± 1.0 b	2.0 ± 0.5 b	146.1 ± 37.2
BfH	4.9 ± 0.9 b	1.9 ± 0.3 b	133.5 ± 35.4
ANOVA statistics	n = 12, F_2, 9_ = 18.23, ***p*** = **0.0007**	n = 12, F_2, 9_ = 6.51, ***p*** = **0.0178**	n = 12, F_2, 9_ = 0.2233, *p* = 0.8041

Root galling index scale (0—no infestation, 10—completely infested). Data presented as averages with the standard deviation (±SD); ANOVA statistic (n—dataset size, F_df, df_—F-statistic with degrees of freedom (df) between groups, and df within groups; *p*—*p*-value); and Tukey’s HSD results. Means sharing a letter are not significantly different at *p* < 0.05. NC—negative control; PC—positive control (nematicide); BfH—high *B. firmus* inoculum.

**Table 3:** Plant morphology parameters at 48 days after inoculation (DAI) in different treatments in the pot experiment.

Treatment	Plant Height (cm)	Leaf Area (cm^2^)	Plant Dry Weight (g)	Root Fresh Weight (g)
NC	111.0 ± 9.6	2213.1 ± 198.2 a	17.1 ± 2.0 a	41.3 ± 4.2 a
PC	108.1 ± 10.7	2765.9 ± 242.6 b	22.8 ± 1.5 b	36.6 ± 3.3 a
BfL	109.6 ± 9.1	3503.4 ± 149.6 c	23.7 ± 0.9 bc	47.4 ± 6.3 a
BfH	104.1 ± 6.3	3719.7 ± 110.5 c	26.2 ± 0.6 c	58.9 ± 6.6 b
ANOVA statistics	n = 16, F_3, 12_ = 0.43, *p* = 0.7343	n = 16, F_3, 12_ = 57.67, ***p*** < **0.0001**	n = 16, F_3, 12_ = 32.42, ***p*** < **0.0001**	n = 16, F_3, 12_ = 13.35, ***p*** = **0.0004**

Data presented as averages with the standard deviation (±SD); ANOVA statistic (n—dataset size, F_df, df_—F-statistic with degrees of freedom (df) between groups, and df within groups; *p*—*p*-value); and Tukey’s HSD results. Means sharing a letter are not significantly different at *p* < 0.05. NC—negative control; PC—positive control (nematicide); BfL—low *B. firmus* inoculum; BfH—high *B. firmus* inoculum.

**Table 4:** Physiological parameters assessing plant photosynthesis and chlorophyll *a* fluorescence at the end of both experiments with different treatments.

Treatment	Photosynthesis Rate (μmol CO_2_ m^−2^ s^−1^)	Stomatal Conductance (mol H_2_O m^−2^ s^−1^)	Transpiration (mmol H_2_O m^−2^ s^−1^)	Effective Quantum Yield of PSII	ETR (μmol e-m^−2^ s^−1^)	Fv/Fm
*Pot experiment*:						
NC	4.90 ± 2.87	0.20 ± 0.16	3.26 ± 2.29	0.47 ± 0.14 a	39.96 ± 12.91 a	0.74 ± 0.048 a
PC	7.99 ± 1.18	0.20 ± 0.05	3.34 ± 0.81	0.73 ± 0.03 b	61.14 ± 3.80 b	0.80 ± 0.005 b
BfL	8.43 ± 2.44	0.19 ± 0.09	3.42 ± 1.57	0.76 ± 0.01 b	69.04 ± 4.06 b	0.79 ± 0.002 b
BfH	8.83 ± 0.95	0.22 ± 0.06	3.89 ± 0.89	0.75 ± 0.03 b	67.03 ± 7.18 b	0.80 ± 0.009 b
ANOVA statistics	n = 16, F_3, 12_ = 3.12, *p* = 0.0663	n = 16, F_3, 6.38_ = 0.08, *p* = 0.9667	n = 16, F_3, 12_ = 0.14, *p* = 0.932	n = 80, F_3, 76_ = 58.62, ***p*** < **0.0001**	n = 16, F_3, 12_ = 11.39, ***p*** = **0.0008**	n = 32, F_3, 13.96_ = 4.41, ***p* = 0.0221**
*Microplot experiment*:						
NC	15.48 ± 1.57	0.21 ± 0.03	3.17 ± 0.30	0.34 ± 0.08 a	124.57 ± 15.94	0.72 ± 0.05 a
PC	11.34 ± 5.17	0.12 ± 0.07	2.13 ± 1.02	0.36 ± 0.15 ab	119.35 ± 48.36	0.76 ± 0.03 ab
BfH	17.62 ± 2.99	0.20 ± 0.05	3.34 ± 0.54	0.48 ± 0.19 b	145.31 ± 19.09	0.78 ± 0.04 b
ANOVA statistics	n = 12, F_2, 9_ = 3.21, *p* = 0.0887	n = 12, F_2, 9_ = 3.51, *p* = 0.0748	n = 12, F_2, 9_ = 3.61, *p* = 0.0706	n = 60, F_2, 33.65_ = 4.19, ***p* = 0.0237**	n = 12, F_2, 9_ = 3.91, *p* = 0.493	n = 24, F_2, 21_ = 4.17, ***p*** = **0.02977**

Data presented as averages with the standard deviation (±SD); ANOVA statistic (n—dataset size, F_df, df_—F-statistic with degrees of freedom (df) between groups, and df within groups; *p*—*p*-value); and Tukey’s HSD results. Means sharing a letter are not significantly different at *p* < 0.05. NC—negative control; PC—positive control (nematicide); BfL—low *B. firmus* inoculum; BfH—high *B. firmus* inoculum.

**Table 5:** Nutrient composition of tomato leaves in different treatments in the microplot experiment.

Element/Parameter	Concentration in Different Treatments			ANOVA Statistics
	NC	PC	BfH	
Relative chlorophyll content	46.12 ± 2.07 a	49.89 ± 4.23 a	59.72 ± 6.42 b	F_2, 9_ = 9.34, ***p*** = **0.0064**
N, %	2.48 ± 0.46 a	1.82 ± 0.26 b	2.77 ± 0.23 a	F_2, 9_ = 8.57, ***p*** = **0.0083**
P, %	0.24 ± 0.05	0.19 ± 0.06	0.20 ± 0.01	F_2, 9_ = 1.68, *p* = 0.2401
K, %	2.07 ± 0.51	1.70 ± 0.41	2.27 ± 0.20	F_2, 9_ = 2.16, *p* = 0.1708
Ca, %	3.74 ± 0.75 a	3.02 ± 0.72 ab	2.18 ± 0.27 b	F_2, 9_ = 6.34, ***p*** = **0.0191**
Mg, %	0.55 ± 0.17	0.57 ± 0.13	0.55 ± 0.09	F_2, 9_ = 0.04, *p* = 0.9616
S, %	0.77 ± 0.14 a	0.65 ± 0.21 ab	0.41 ± 0.04 b	F_2, 9_ = 6.39, ***p*** = **0.0188**
Na, µg g^−1^	496.25 ± 199.16	460.35 ± 246.00	751.68 ± 193.44	F_2, 9_ = 2.20, *p* = 0.1667
Cu, µg g^−1^	8.84 ± 1.43 a	5.40 ± 1.09 b	8.62 ± 1.05 a	F_2, 9_ = 10.33, ***p*** = **0.0047**
Fe, µg g^−1^	145.44 ± 21.49	155.10 ± 48.69	125.73 ± 23.32	F_2, 9_ = 0.80, *p* = 0.4803
Mn, µg g^−1^	49.60 ± 17.34	44.83 ± 11.31	41.95 ± 10.76	F_2, 9_ = 0.33, *p* = 0.7279
Mo, µg g^−1^	3.78 ± 0.91 a	3.16 ± 0.84 ab	2.21 ± 0.21 b	F_2, 9_ = 4.75, ***p*** = **0.0391**
Zn, µg g^−1^	19.82 ± 3.29 ab	16.15 ± 1.56 a	23.14 ± 2.87 b	F_2, 9_ = 6.83, ***p*** = **0.0157**

Dataset size for each analysis, n = 12. Data presented as averages with the standard deviation (±SD); ANOVA statistic (F_df, df_—F-statistic with degrees of freedom (df) between groups, and df within groups; *p*—*p*-value); and Tukey’s HSD results. Means sharing a letter are not significantly different at *p* < 0.05. NC—negative control; PC—positive control (nematicide); BfH—high *B. firmus* inoculum.

**Table 6:** Summary of the partial least squares discriminant analysis (PLS-DA) and partial least squares support vector machines (PLS-SVM) analysis. The described datasets were also used in principal component analysis (PCA).

Analysis	Treatments	Dataset Size	PLS-DA		SVM		Accuracy (%)		Confusion Matrix
			Var (%)	RMSECV	c	Gamma	Ts	CV	
*Pot experiment*:									
Treatment—pooled	BfL:BfH:PC:NC	119	32.50	0.356	0.34	0.03	96.3	87.2	Table S6
Untreated vs. treated	NC:PC, BfL, BfH	119	82.16	0.192	0.01	0.01	100	100	Table S7
*B. firmus*-inoculated vs. non-inoculated	BfL, BfH:NC, PC	119	56.50	0.319	0.01	0.02	100	97.4	Table S8
*B. firmus* inoculum size	BfL:BfH	64	55.44	0.274	0.01	0.01	100	100	Table S9
*Microplot experiment*:									
Treatment—pooled	BfH:PC:NC	82	79.10	0.298	0.24	0.02	98.9	96.3	Table S10

Var—explained variance of the selected PLS components; RMSECV—root mean squared error of cross-validation of selected PLS components; c—SVM cost of classification parameter; gamma—SVM Gaussian kernel parameter; Ts—training set; CV—cross-validation; NC—negative control; PC—positive control (nematicide); BfL—low *B. firmus* inoculum; BfH—high *B. firmus* inoculum.

**Table 7:** Experimental design of the pot and microplot experiments and treatment group designations.

Groups	Pot Experiment Treatments	Microplot Experiment Treatments	Treatment Description
Without added bacteria (Bf−)	NC	NC	Untreated tomato plants infested with RKN (negative control)
	PC	PC	Plants infested with RKN and treated with the chemical nematicide Velum Prime (positive control)
With added bacteria (Bf+)	BfL	n/a	Plants infested with RKN and treated with the biological nematicide VOTiVO—seed coating only (Low *B. firmus* inoculum)
	BfH	BfH	Plants infested with RKN and treated with the biological nematicide VOTiVO—seed coating and substrate drenching (High *B. firmus* inoculum)

Bf—*Bacillus firmus*; n/a—treatment not performed.

## Supplementary Materials

The following are available online at https://www.mdpi.com/2223-7747/9/5/592/s1, Table S1: npMANOVA results on pot experiment, microplot experiment and leaf-nutrient datasets, Table S2: Pairwise dissimilarity tests of the pot experiment data, Table S3: Pairwise dissimilarity tests of the microplot experiment data, Table S4: Pairwise dissimilarity tests of leaf tissue nutrient composition data, Table S5: PLS-R model quality evaluation, Table S6: PLS-SVM classification confusion matrix for distinguishing among all treatment groups in the pot experiment, Table S7: PLS-SVM classification confusion matrix for distinguishing between treated and untreated plants in the pot experiment, Table S8: PLS-SVM classification confusion matrix for distinguishing between *B. firmus*-inoculated and non-inoculated plants in the pot experiment, Table S9: PLS-SVM classification confusion matrix for distinguishing between plants with low and high levels of *B. firmus* inoculation in the pot experiment, Table S10: PLS-SVM classification confusion matrix for distinguishing among all treatment groups in the microplot experiment, Figure S1: PCA biplot of the measured variables in the pot experiment, Figure S2: PCA biplot of the microplot experiment data, Figure S3: PCA biplot of the leaf nutrient composition data.

## References

[1] Carter C.C., Sasser J.N. (1985). Overview of the International Meloidogyne Project 1975–1984. An Advanced Treatise on Meloidogyne, Volume I: Biology and Control.

[2] Fuller V.L., Lilley C.J., Urwin P.E. (2008). Nematode resistance. New Phytol..

[3] Strajnar P., Širca S., Urek G., Šircelj H., Železnik P., Vodnik D. (2012). Effect of Meloidogyne ethiopica parasitism on water management and physiological stress in tomato. Eur. J. Plant Pathol..

[4] Janssen T., Karssen G., Verhaeven M., Coyne D., Bert W. (2016). Mitochondrial coding genome analysis of tropical root-knot nematodes (*Meloidogyne*) supports haplotype based diagnostics and reveals evidence of recent reticulate evolution. Sci. Rep..

[5] Gerič Stare B., Strajnar P., Susič N., Urek G., Širca S. (2017). Reported populations of *Meloidogyne ethiopica* in Europe identified as *Meloidogyne luci*. Plant Dis..

[6] Chitwood D.J. (2002). Phytochemical Based Strategies for Nematode Control. Annu. Rev. Phytopathol..

[7] Tian B., Yang J., Zhang K.-Q. (2007). Bacteria used in the biological control of plant-parasitic nematodes: Populations, mechanisms of action, and future prospects. FEMS Microbiol. Ecol..

[8] Wilson M.J., Jackson T.A. (2013). Progress in the commercialisation of bionematicides. BioControl.

[9] Sukumaran S., Dailin D.J., Malek R.A., Peng T., Hanapi S.Z., Enshasy H.E. (2019). Production of High Cell Mass of Bacillus firmus Using Statistical Medium Optimization. J. Sci. Ind. Res..

[10] Geng C., Nie X., Tang Z., Zhang Y., Lin J., Sun M., Peng D. (2016). A novel serine protease, Sep1, from *Bacillus firmus* DS-1 has nematicidal activity and degrades multiple intestinal-associated nematode proteins. Sci. Rep..

[11] Lian L.H., Tian B.Y., Xiong R., Zhu M.Z., Xu J., Zhang K.Q. (2007). Proteases from *Bacillus*: A new insight into the mechanism of action for rhizobacterial suppression of nematode populations. Lett. Appl. Microbiol..

[12] Susič N., Janežič S., Rupnik M., Gerič Stare B. (2020). Whole Genome Sequencing and Comparative Genomics of Two Nematicidal *Bacillus* Strains Reveals a Wide Range of Possible Virulence Factors. G3.

[13] Keren-Zur M., Antonov J., Bercovitz A., Feldman K., Husid A., Kenan G., Markov N., Rebhun M. *Bacillus firmus* Formulations for the Safe Control of Root-Knot Nematodes. Proceedings of the Brighton Crop Protection Conference on Pests and Diseases.

[14] Giannakou I.O., Karpouzas D.G., Prophetou-Athanasiadou D. (2004). A novel non-chemical nematicide for the control of root-knot nematodes. Appl. Soil Ecol..

[15] Giannakou I.O., Anastasiadis I.A., Gowen S.R., Prophetou-Athanasiadou D.A. (2007). Effects of a non-chemical nematicide combined with soil solarization for the control of root-knot nematodes. Crop Prot..

[16] Terefe M., Tefera T., Sakhuja P.K. (2009). Effect of a formulation of *Bacillus firmus* on root-knot nematode *Meloidogyne incognita* infestation and the growth of tomato plants in the greenhouse and nursery. J. Invertebr. Pathol..

[17] Mendoza A.R., Kiewnick S., Sikora R.A. (2008). In vitro activity of *Bacillus firmus* against the burrowing nematode *Radopholus similis*, the root-knot nematode *Meloidogyne incognita* and the stem nematode *Ditylenchus dipsaci*. Biocontrol Sci. Technol..

[18] Crow W.T. (2014). Effects of a Commercial Formulation of *Bacillus firmus* I-1582 on Golf Course Bermudagrass Infested with *Belonolaimus longicaudatus*. J. Nematol..

[19] Terefe M., Tefera T., Sakhuja P.K. (2012). Biocontrol (Formulation of Bacillus firmus (BioNem)) of Root-knot Nematode, Meloidogyne incognita on Tomato Plants in the Field. Ethiop. J. Agric. Sci..

[20] Goswami D., Thakker J.N., Dhandhukia P.C. (2016). Portraying mechanics of plant growth promoting rhizobacteria (PGPR): A review. Cogent Food Agric..

[21] Abbasi M.W., Ahmed N., Zaki M.J., Shuakat S.S., Khan D. (2014). Potential of *Bacillus* species against *Meloidogyne javanica* parasitizing eggplant (*Solanum melongena* L.) and induced biochemical changes. Plant Soil.

[22] El-Esawi M.A., Alaraidh I.A., Alsahli A.A., Alamri S.A., Ali H.M., Alayafi A.A. (2018). *Bacillus firmus* (SW5) augments salt tolerance in soybean (*Glycine max* L.) by modulating root system architecture, antioxidant defense systems and stress-responsive genes expression. Plant Physiol. Biochem..

[23] Adesemoye A.O., Kloepper J.W. (2009). Plant–microbes interactions in enhanced fertilizer-use efficiency. Appl. Microbiol. Biotechnol..

[24] Reyns P., Missotten B., Ramon H., De Baerdemaeker J. (2002). A Review of Combine Sensors for Precision Farming. Precis. Agric..

[25] McBratney A., Whelan B., Ancev T., Bouma J. (2005). Future Directions of Precision Agriculture. Precis. Agric..

[26] Sankaran S., Mishra A., Ehsani R., Davis C. (2010). A review of advanced techniques for detecting plant diseases. Comput. Electron. Agric..

[27] Huang H., Liu L., Ngadi M. (2014). Recent Developments in Hyperspectral Imaging for Assessment of Food Quality and Safety. Sensors.

[28] Brugger A., Behmann J., Paulus S., Luigs H.-G., Kuska M.T., Schramowski P., Kersting K., Steiner U., Mahlein A.-K. (2019). Extending Hyperspectral Imaging for Plant Phenotyping to the UV-Range. Remote Sens..

[29] Susič N., Žibrat U., Širca S., Strajnar P., Razinger J., Knapič M., Vončina A., Urek G., Gerič Stare B. (2018). Discrimination between abiotic and biotic drought stress in tomatoes using hyperspectral imaging. Sens. Actuators B Chem..

[30] Zhao Y.-R., Li X., Yu K.-Q., Cheng F., He Y. (2016). Hyperspectral Imaging for Determining Pigment Contents in Cucumber Leaves in Response to Angular Leaf Spot Disease. Sci. Rep..

[31] Carvalho S., van der Putten W.H., Hol W.H.G. (2016). The Potential of Hyperspectral Patterns of Winter Wheat to Detect Changes in Soil Microbial Community Composition. Front. Plant Sci..

[32] Elsayed S., Elhoweity M., El-Hendawy S., Schmidhalter U. (2017). Non-invasive spectral detection of the beneficial effects of *Bradyrhizobium* spp. and plant growth-promoting rhizobacteria under different levels of nitrogen application on the biomass, nitrogen status, and yield of peanut cultivars. Bragantia.

[33] Dahlin P., Eder R., Consoli E., Krauss J., Kiewnick S. (2019). Integrated control of *Meloidogyne incognita* in tomatoes using fluopyram and *Purpureocillium lilacinum* strain 251. Crop Prot..

[34] Beeman A.Q., Njus Z.L., Pandey S., Tylka G.L. (2019). The Effects of ILeVO and VOTiVO on Root Penetration and Behavior of the Soybean Cyst Nematode, *Heterodera glycines*. Plant Dis..

[35] Susič N., Širca S., Strajnar P., Gerič Stare B. Assessing the Nematicidal Activity of *Bacillus firmus* Strains. Proceedings of the 14th Slovenian Conference on Plant Protection with International Participation; Plant Protection Society of Slovenia—Ljubljana.

[36] Musil K.M. (2016). Evaluations of Biological Control Agents for the Management of Soybean Cyst Nematode (Heterodera glycines) in Soybean (Glycine max L. Merr.).

[37] Beeman A.Q., Tylka G.L. (2018). Assessing the Effects of ILeVO and VOTiVO Seed Treatments on Reproduction, Hatching, Motility, and Root Penetration of the Soybean Cyst Nematode, *Heterodera glycines*. Plant Dis..

[38] Adam G., Duncan H. (2001). Development of a sensitive and rapid method for the measurement of total microbial activity using fluorescein diacetate (FDA) in a range of soils. Soil Biol. Biochem..

[39] Duca D., Lorv J., Patten C.L., Rose D., Glick B.R. (2014). Indole-3-acetic acid in plant–microbe interactions. Antonie Van Leeuwenhoek.

[40] Shi H., Wang L., Li X., Liu X., Hao T., He X., Chen S. (2016). Genome-wide transcriptome profiling of nitrogen fixation in *Paenibacillus* sp. WLY78. BMC Microbiol..

[41] Pajares S., Bohannan B.J.M. (2016). Ecology of Nitrogen Fixing, Nitrifying, and Denitrifying Microorganisms in Tropical Forest Soils. Front. Microbiol..

[42] Hendriks J., Oubrie A., Castresana J., Urbani A., Gemeinhardt S., Saraste M. (2000). Nitric oxide reductases in bacteria. Biochim. Biophys. Acta Bioenerg..

[43] Luque-Almagro V.M., Gates A.J., Moreno-Vivián C., Ferguson S.J., Richardson D.J., Roldán M.D. (2011). Bacterial nitrate assimilation: Gene distribution and regulation. Biochem. Soc. Trans..

[44] Bu C., Wang Y., Ge C., Ahmad H.A., Gao B., Ni S.-Q. (2017). Dissimilatory Nitrate Reduction to Ammonium in the Yellow River Estuary: Rates, Abundance, and Community Diversity. Sci. Rep..

[45] Calvo P., Zebelo S., McNear D., Kloepper J., Fadamiro H. (2019). Plant growth-promoting rhizobacteria induce changes in *Arabidopsis thaliana* gene expression of nitrate and ammonium uptake genes. J. Plant Interact..

[46] Zeng Q., Xie J., Li Y., Gao T., Xu C., Wang Q. (2018). Comparative genomic and functional analyses of four sequenced *Bacillus cereus* genomes reveal conservation of genes relevant to plant-growth-promoting traits. Sci. Rep..

[47] Meena V.S., Bahadur I., Maurya B.R., Kumar A., Meena R.K., Meena S.K., Verma J.P., Meena V.S., Maurya B.R., Verma J.P., Meena R.S. (2016). Potassium-Solubilizing Microorganism in Evergreen Agriculture: An Overview. Potassium Solubilizing Microorganisms for Sustainable Agriculture.

[48] Adams P., Atherton J.G., Rudich J. (1986). Mineral Nutrition. The Tomato Crop: A Scientific Basis for Improvement.

[49] Wilson M.K., Abergel R.J., Raymond K.N., Arceneaux J.E.L., Byers B.R. (2006). Siderophores of Bacillus anthracis, Bacillus cereus, and Bacillus thuringiensis. Biochem. Biophys. Res. Commun..

[50] Wilson M.K., Abergel R.J., Arceneaux J.E.L., Raymond K.N., Byers B.R. (2010). Temporal Production of the Two *Bacillus anthracis* Siderophores, Petrobactin and Bacillibactin. Biometals.

[51] Maathuis F.J.M. (2014). Sodium in plants: Perception, signalling, and regulation of sodium fluxes. J. Exp. Bot..

[52] Pan J., Peng F., Xue X., You Q., Zhang W., Wang T., Huang C. (2019). The Growth Promotion of Two Salt-Tolerant Plant Groups with PGPR Inoculation: A Meta-Analysis. Sustainability.

[53] Croft H., Chen J.M. (2018). Leaf Pigment Content. Comprehensive Remote Sensing.

[54] Liu C., Guo J., Cui Y., Lü T., Zhang X., Shi G. (2011). Effects of cadmium and salicylic acid on growth, spectral reflectance and photosynthesis of castor bean seedlings. Plant Soil.

[55] Ryu C.-M., Murphy J.F., Mysore K.S., Kloepper J.W. (2004). Plant growth-promoting rhizobacteria systemically protect *Arabidopsis thaliana* against *Cucumber mosaic virus* by a salicylic acid and NPR1-independent and jasmonic acid-dependent signaling pathway. Plant J..

[56] Fan J.W., Hu C.L., Zhang L.N., Li Z.L., Zhao F.K., Wang S.H. (2015). Jasmonic Acid Mediates Tomato’s Response to Root Knot Nematodes. J. Plant Growth Regul..

[57] Kaya A., Doganlar Z.B. (2016). Exogenous jasmonic acid induces stress tolerance in tobacco (*Nicotiana tabacum*) exposed to imazapic. Ecotoxicol. Environ. Saf..

[58] Gitelson A.A., Gritz Y., Merzlyak M.N. (2003). Relationships between leaf chlorophyll content and spectral reflectance and algorithms for non-destructive chlorophyll assessment in higher plant leaves. J. Plant Physiol..

[59] Wang Q., Chen J., Stamps R.H., Li Y. (2005). Correlation of Visual Quality Grading and SPAD Reading of Green-Leaved Foliage Plants. J. Plant Nutr..

[60] Zhang L., Zhou Z., Zhang G., Meng Y., Chen B., Wang Y. (2012). Monitoring the leaf water content and specific leaf weight of cotton (*Gossypium hirsutum* L.) in saline soil using leaf spectral reflectance. Eur. J. Agron..

[61] Elvanidi A., Katsoulas N., Ferentinos K.P., Bartzanas T., Kittas C. (2018). Hyperspectral machine vision as a tool for water stress severity assessment in soilless tomato crop. Biosyst. Eng..

[62] Yue J., Tian Q., Dong X., Xu K., Zhou C. (2019). Using Hyperspectral Crop Residue Angle Index to Estimate Maize and Winter-Wheat Residue Cover: A Laboratory Study. Remote Sens..

[63] Kokaly R.F. (2001). Investigating a Physical Basis for Spectroscopic Estimates of Leaf Nitrogen Concentration. Remote Sens. Environ..

[64] Thenkabail P.S., Mariotto I., Gumma M.K., Middleton E.M., Landis D.R., Huemmrich K.F. (2013). Selection of Hyperspectral Narrowbands (HNBs) and Composition of Hyperspectral Twoband Vegetation Indices (HVIs) for Biophysical Characterization and Discrimination of Crop Types Using Field Reflectance and Hyperion/EO-1 Data. IEEE J. Sel. Top Appl. Earth Obs. Remote Sens..

[65] Kokaly R.F., Skidmore A.K. (2015). Plant phenolics and absorption features in vegetation reflectance spectra near 1.66 μm. Int. J. Appl. Earth Obs. Geoinf..

[66] Xiong D., Chen J., Yu T., Gao W., Ling X., Li Y., Peng S., Huang J. (2015). SPAD-based leaf nitrogen estimation is impacted by environmental factors and crop leaf characteristics. Sci. Rep..

[67] Susič N., Koutsovoulos G.D., Riccio C., Danchin É.G.J., Blaxter M.L., Lunt D.H., Strajnar P., Širca S., Urek G., Gerič Stare B. (2020). Genome sequence of the root-knot nematode *Meloidogyne luci*. J. Nematol..

[68] Razinger J., Lutz M., Grunder J., Urek G. (2018). Laboratory Investigation of Cauliflower–Fungus–Insect Interactions for Cabbage Maggot Control. J. Econ. Entomol..

[69] Zeck W.M. (1971). A rating scheme for field evaluation of root-knot nematode infestations. Pflanzenschutz-Nachr. Bayer.

[70] Urek G., Hržič A., Urek G. (1998). Nematodes—Invisible Plant Parasites (Phytonematology).

[71] Haupt-Herting S., Klug K., Fock H.P. (2001). A new approach to measure gross CO_2_ fluxes in leaves. Gross CO_2_ assimilation, photorespiration, and mitochondrial respiration in the light in tomato under drought stress. Plant Physiol..

[72] Žibrat U., Susič N., Knapič M., Širca S., Strajnar P., Razinger J., Vončina A., Urek G., Gerič Stare B. (2019). Pipeline for imaging, extraction, pre-processing, and processing of time-series hyperspectral data for discriminating drought stress origin in tomatoes. MethodsX.

[73] Földes T., Bánhegyi I., Herpai Z., Varga L., Szigeti J. (2000). Isolation of *Bacillus* strains from the rhizosphere of cereals and in vitro screening for antagonism against phytopathogenic, food-borne pathogenic and spoilage micro-organisms. J. Appl. Microbiol..

[74] Agrawal D.P.K., Agrawal S. (2013). Characterization of *Bacillus* sp. strains isolated from rhizosphere of tomato plants (*Lycopersicon esculentum*) for their use as potential plant growth promoting rhizobacteria. Int. J. Curr. Microbiol. Appl. Sci..

[75] Mendis H.C., Thomas V.P., Schwientek P., Salamzade R., Chien J.-T., Waidyarathne P., Kloepper J., Fuente L.D.L. (2018). Strain-specific quantification of root colonization by plant growth promoting rhizobacteria *Bacillus firmus* I-1582 and *Bacillus amyloliquefaciens* QST713 in non-sterile soil and field conditions. PLoS ONE.

[76] The UniProt Consortium (2015). UniProt: A hub for protein information. Nucleic Acids Res..

[77] Bressy F.C., Brito G.B., Barbosa I.S., Teixeira L.S.G., Korn M.G.A. (2013). Determination of trace element concentrations in tomato samples at different stages of maturation by ICP OES and ICP-MS following microwave-assisted digestion. Microchem. J..

[78] Muñoz-Huerta R.F., Guevara-Gonzalez R.G., Contreras-Medina L.M., Torres-Pacheco I., Prado-Olivarez J., Ocampo-Velazquez R.V. (2013). A Review of Methods for Sensing the Nitrogen Status in Plants: Advantages, Disadvantages and Recent Advances. Sensors.

[79] R Core Team (2018). R: A Language and Environment for Statistical Computing.

[80] Oksanen J., Blanchet F.G., Friendly M., Kindt R., Legendre P., McGlinn D., Minchin P.R., O’Hara R.B., Simpson G.L., Solymos P. (2019). Vegan: Community Ecology Package.

[81] Vu V. (2015). ggbiplot: An Implementation of the Biplot Using ggplot2.

[82] RStudio Team (2016). RStudio: Integrated Development for R.

[83] Wickham H. (2016). ggplot2: Elegant Graphics for Data Analysis.

[84] Barter R.L., Yu B. (2018). Superheat: An R package for creating beautiful and extendable heatmaps for visualizing complex data. J. Comput. Graph. Stat..

