# Supplementary material for: From Genome to Field—Observation of the Multimodal Nematicidal and Plant Growth-Promoting Effects of Bacillus firmus I-1582 on Tomatoes Using Hyperspectral Remote Sensing
Source: Plants (Basel). 2020 May 6;9(5):592. doi: 10.3390/plants9050592 (PMC7285481; doi:10.3390/plants9050592)
Supplement: Supplementary file 1 [file plants-09-00592-s001.pdf]

plants MDPI

Supplementary Material

From Genome to Field—Observation of the Multimodal Nematicidal and Plant Growth-Promoting Effects of *Bacillus firmus* I-1582 on Tomatoes Using Hyperspectral Remote Sensing

Nik Susič, Uroš Žibrat, Lovro Sinkovič, Andrej Vončina, Jaka Razinger, Matej Knapič, Aleš Sedlar, Saša Širca, Barbara Gerič Stare

1. Supplementary Results and Discussion

1.1. Exploratory Data Analysis

The data obtained for different plant physiology variables (stomatal conductance, ETR, F_v/F_m, photosynthesis rate, transpiration and the effective quantum yield of PSII), morphology (plant dry biomass, number of flowers per plant, plant height, total leaf area per plant and fresh root weight), nutrition (concentration of various nutrients in leaf tissue), microbial activity (fluorescein release (g^{-1} substrate)), and nematode reproduction (number of nematode eggs per plant, R_f and galling index) in the pot and microplot experiment datasets were explored with permutational (non-parametric) multivariate analysis of variance (npMANOVA) and principal component analysis (PCA). npMANOVA showed there were significant differences between the treatments for pot, microplot and nutrient content (from microplot samples) datasets at $p < 0.05$ (Table S1 a–c). Further pairwise comparison of treatments in the pot experiment showed that NC, PC and *Bacillus*-treated plants differed from each other, but the differences between BfL and BfH were not statistically significant (Table S2). Analysis showed that a lower or higher initial bacterial inoculum did not result in discernable effects on the measured parameters included in the analysis (see Methods). Pairwise npMANOVA showed that the plant parameters differed for NC, PC and BfH treatments in the microplot experiment data, while the differences between the PC and BfH were not significant (Table S3). In contrast, analysis of nutrient abundance and distribution showed significant differences between the NC and BfH, as well as the PC and BfH, but not between the NC and PC (Table S4). Considering npMANOVA alone, *B. firmus* treatment had significant effects on the nutrient composition of plants in the microplot experiment.

PCA was carried out with the same three datasets as for npMANOVA, using 14 selected variables from the pot experiment (Figure S1), and 11 selected variables from the microplot experiment (Figure S2) and nutrient content dataset (Figure S3). PCA was used to describe the main factors contributing to variance between the treatment groups and was conducted in R [1] with the software package 'ggbiplot' (v0.55) [2]. PCA was mostly successful in separating samples in all three datasets according to treatment groups using the first two principal components, and the separation corresponded with hierarchical clustering analysis. PCA of the pot experiment dataset showed well-separated individuals according to NC, PC and *Bacillus*-treated plant treatment groups, while different *B. firmus* inoculum groups (BfL and BfH) were not separated (Figure S1). Clustering analysis corresponded with PCA, the exception being one aberrant individual from BfL that clustered with the PC. Based on variable loadings on the first factorial plane, NC plants were most influenced by higher values (and the PC cluster by lower values) of nematode reproduction parameters (number of nematode eggs, R_f). High values for some of the plant physiological parameters (photosynthesis rate, ETR, F_v/F_m and effective quantum yield of PSII) and morphological parameters (root weight, leaf area and plant biomass) had the greatest influence on the group of *B. firmus*-treated plants. Leaf area, plant

biomass, number of flowers and fluorescein release parameters were most negatively correlated with nematode reproduction parameters. The results indicated some positive effects of *B. firmus* on plant morphology and physiology, despite not eliminating the RKN infestation completely (as in the case of the PC). In contrast to the controlled environment of the pot experiment, the analogous open-field microplot environment introduced more variability to the data, now predominantly affecting the PC group, as seen in the PCA biplot (Figure S2). Hierarchical clustering determined 3 groups according to the treatments, the exception being one sample from the PC that clustered with the NC. As with the pot experiment dataset, samples from BfH were most associated with higher values for the effective quantum yield of PSII, F_v/F_m, and high relative chlorophyll content, while the NC cluster was associated with a high root galling index and high nematode counts in the soil. The PC cluster was associated with low values for transpiration and stomatal conductance, rather than with low values for nematode reproduction parameters, due to the chemical nematicide being less effective in the microplot experiment compared to the pot experiment. PCA on the nutrient dataset also showed differences between samples in all 3 treatments (Figure S3). The samples clustered together into 3 groups according to treatments, although one NC sample clustered with the PC and one PC sample clustered more readily with the NC. All the BfH samples clustered together. Variable loadings on the first factorial plane indicated a complex influence of various nutrients on samples from the three treatment groups. The NC was influenced by higher phosphorous values (P), the PC mostly by lower potassium (K), nitrogen (N) and copper (Cu) values, and BfH was influenced by high relative chlorophyll content, lower Calcium (Ca), molybdenum (Mo), sulphur (S) and to a lesser extent iron (Fe) and magnesium (Mg) values.

2. Supplementary Tables

2.1. Permutational Multivariate Analysis of Variance (npMANOVA)

Table S1. npMANOVA results based on Euclidean dissimilarities using measurement of various plant physiology, morphology, nutrition and nematode reproduction variables in relation to the treatments for a) pot experiment (n = 16), b) microplot (n = 12), and c) leaf-nutrient (n = 12) datasets. Analysis was conducted in R using the 'adonis' function in the 'vegan' (v2.5-6) package [3].

Experiment dataset	Df	SS	F-model	R^2	p
**(a) pot experiment**					
treatment	3	0.712	7.986	0.666	**0.001**
residuals	12	0.357		0.333	
total	5	1.069		1.000	
**b) microplot**					
treatment	2	0.308	2.972	0.398	**0.003**
residuals	9	0.467		0.602	
total	11	0.775		1.000	
**c) leaf-nutrients**					
treatment	2	0.570	3.573	0.443	**0.003**
residuals	9	0.717		0.557	
total	11	1.287		1.000	

Df – degrees of freedom; SS – sum of squares; F-model – F value by permutation; R^2 – coefficient of determination; p – p-values based on 999 permutations (value in bold indicates statistical significance at $p < 0.05$).

Table S2. Pairwise dissimilarity tests of recorded variables in different treatments in the pot experiment using npMANOVA. R^2 and p-values (brackets) are shown. Analysis was conducted in R using the 'adonis' function in the 'vegan' package (v2.5-6) [3].

	NC	PC	BfL	BfH
**NC**	-	0.550 (**0.034**)	0.502 (**0.028**)	0.614 (**0.033**)
**PC**		-	0.431 (**0.034**)	0.656 (**0.029**)
**BfL**			-	0.182 (0.339)
**BfH**				-

Dataset size, n = 16; NC – negative control; PC – positive control (nematicide); BfL – Low *B. firmus* inoculum; BfH – High *B. firmus* inoculum.

Table S3. Pairwise dissimilarity tests of recorded variables in different treatments in the microplot experiment using npMANOVA. R^2 and p-values (brackets) are shown. Analysis was conducted in R using the 'adonis' function in the 'vegan' package (v2.5-6) [3].

	NC	PC	BfH
**NC**	-	0.417 (**0.026**)	0.505 (**0.028**)
**PC**		-	0.363 (0.063)

Dataset size, n = 12; NC – negative control; PC – positive control (nematicide); BfH – High *B. firmus* inoculum.

Table S4. Pairwise dissimilarity tests of leaf tissue nutrient composition in different treatments in the microplot experiment using npMANOVA. R^2 and p-values (brackets) are shown. Analysis was conducted in R using the 'adonis' function in the 'vegan' package (v2.5-6) [3].

	NC	PC	BfH
**NC**	-	0.408 (0.136)	0.414 (**0.026**)
**PC**		-	0.467 (**0.019**)
**BfH**			-

Dataset size, n = 12; NC – negative control; PC – positive control (nematicide); BfH – High *B. firmus* inoculum.

2.2. Partial Least Squares-Regression (PLS-R) Analysis of Hyperspectral and Nutrient Content Data

Table S5. Partial least squares-regression (PLS-R) model quality evaluation. Each model was generated from 12 observations.

Nutrient	Var (%)	R^2c	R^2cv	RMSEC	RMSECV
N	94.89	0.944	0.714	0.117	0.288
P	84.65	0.846	0.434	0.017	0.036
K	86.69	0.544	0.360	0.279	0.361
Ca	91.14	0.963	0.648	0.159	0.538
Mg	89.40	0.963	0.276	0.023	0.110
S	92.10	0.962	0.674	0.038	0.121
Cu	92.80	0.965	0.620	0.350	1.270
Fe	87.70	0.973	0.430	5.110	25.95
Mn	85.90	0.986	0.190	1.445	11.83
Mo	87.50	0.930	0.367	0.238	0.780
Zn	90.34	0.999	0.370	0.030	3.180

Rel. chlorophyll content	90.20	0.880	0.590	2.370	4.860

Var – explained variance of the first 3 PLS components; R^2_C – explained calibration physiological variance; R^2_{CV} – explained cross-validation physiological variance; RMSEC – root mean squared error of calibration; RMSECV – root mean squared error of cross-validation.

2.3. Partial Least Squares-Support Vector Machines (PLS-SVM) Confusion Matrices

Table S6. Partial least squares-support vector machines (PLS-SVM) classification confusion matrix for distinguishing among all four treatment groups (BfL: BfH: PC: NC) in the pot experiment plants.

		Actual				Sensitivity	Specificity	PPV	NPV
		BfL	NC	PC	BfH				
**predicted**	BfL	9	0	0	0	0.75	1	1	0.9
	NC	0	9	0	0	1	1	1	1
	PC	0	0	6	0	0.75	1	1	0.94
	BfH	3	0	2	10	1	0.83	0.67	1
**Classification Accuracy (%)**	87.2								
**Train set size**	80								
**Test set size**	39								
**Treatments**		BfL	NC	PC	BfH				
**Sample sizes**		33	26	29	31				
**Dataset size**	119								

PPV – Positive predictive value; NPV – Negative predictive value; NC – negative control; PC – positive control (nematicide); BfL – Low *B. firmus* inoculum; BfH – High *B. firmus* inoculum.

Table S7. Partial least squares-support vector machines (PLS-SVM) classification confusion matrix for distinguishing between treated and untreated plants (NC: PC, BfL, BfH) in the pot experiment.

		Actual		Sensitivity	Specificity	PPV	NPV
		Treated	Untreated				
**Predicted**	Treated	30	0	1	1	1	1
	Untreated	0	9				
**Classification Accuracy (%)**	100						
**Train set size**	80						
**Test set size**	39						
**Treatments**		Treated	Untreated				
**Sample sizes**		93	26				
**Dataset size**	119						

PPV – Positive predictive value; NPV – Negative predictive value; NC – negative control; PC – positive control (nematicide); BfL – Low *B. firmus* inoculum; BfH – High *B. firmus* inoculum.

Table S8. Partial least squares-support vector machines (PLS-SVM) classification confusion matrix for distinguishing between *Bacillus firmus*-inoculated and non-inoculated plants (BfL, BfH: NC, PC) in the pot experiment.

		Actual		Sensitivity	Specificity	PPV	NPV
		Inoculated	Non-inoculated				
**Predicted**	Inoculated	21	1	1	0.94	0.95	1
	Non-inoculated	0	17				
**Classification Accuracy (%)**	97.4						

**Train set Size**	80		
**Test set size**	39		
**Treatments**		Inoculated	Non-inoculated
**Sample sizes**		64	55
**Dataset size**	119		

PPV – Positive predictive value; NPV – Negative predictive value; NC – negative control; PC – positive control (nematicide); BfL – Low *B. firmus* inoculum; BfH – High *B. firmus* inoculum.

Table S9. Partial least squares-support vector machines (PLS-SVM) classification confusion matrix for distinguishing between plants with low and high levels of *Bacillus firmus* inoculation (BfL: BfH) in the pot experiment.

		Actual		Sensitivity	Specificity	PPV	NPV
		BfL	BfH				
**Predicted**	BfL	10	0	1	1	1	1
	BfH	0	11				
**Classification accuracy (%)**	100						
**Train set size**	43						
**Test set size**	21						
**Treatments**		BfL	BfH				
**Sample sizes**		33	31				
**Dataset size**	64						

PPV – Positive predictive value; NPV – Negative predictive value; BfL – Low *B. firmus* inoculum; BfH – High *B. firmus* inoculum.

Table S10. Partial least squares-support vector machines (PLS-SVM) classification confusion matrix for distinguishing among all three treatment groups (BfH: PC: NC) in the microplot experiment plants.

		Actual			Sensitivity	Specificity	PPV	NPV
		BfH	PC	NC				
	BfH	9	0	1	1	0.94	0.90	1
**Predicted**	PC	0	9	0	1	1	1	1
	NC	0	0	8	0.89	1	1	0.95
**Classification accuracy (%)**	96.3							
**Train set size**	55							
**Test set size**	27							
**Treatments**		BfH	PC	NC				
**Sample sizes**		28	26	28				
**Dataset size**	82							

PPV – Positive predictive value; NPV – Negative predictive value; NC – negative control; PC – positive control (nematicide); BfH – High *B. firmus* inoculum.

3. Supplementary Figures

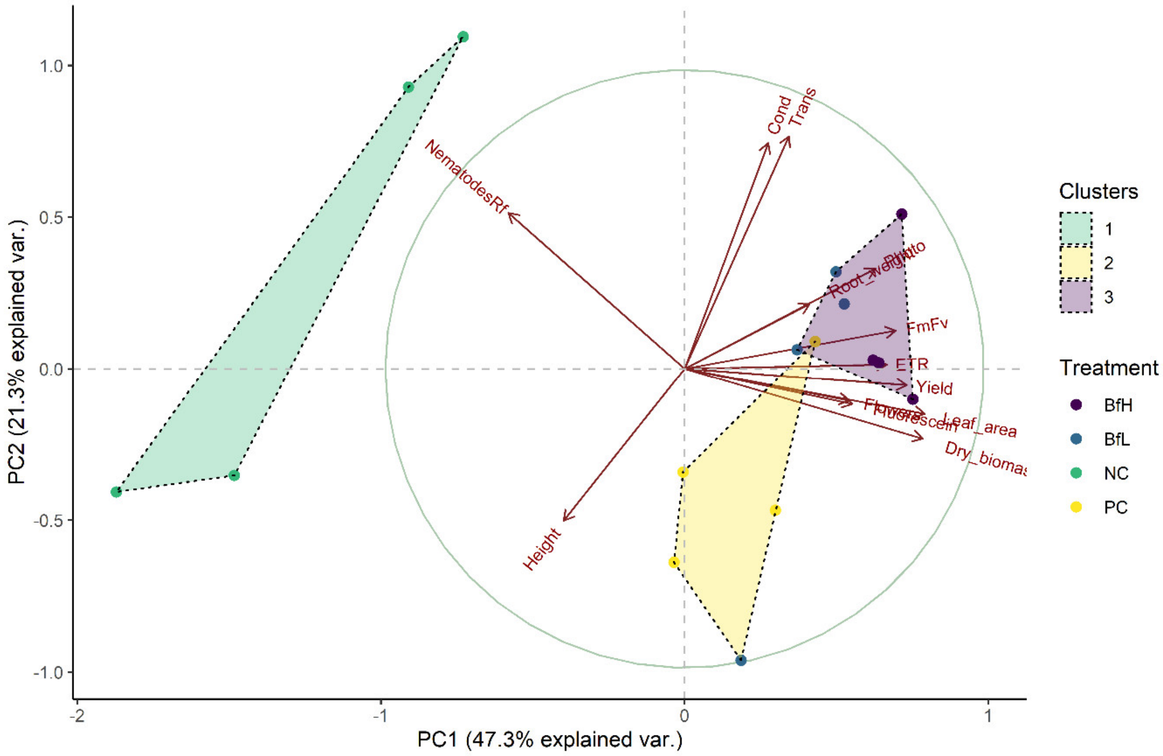

Figure S1. Principal component analysis (PCA) biplot of the 14 measured variables in 16 plants in NC, PC, BfL and BfH treatments in the pot experiment. The variables used in PCA were: Cond – stomatal conductance; Dry_biomass – plant dry biomass; ETR – electron transport rate; Flowers – number of flowers per plant; Fluorescein – fluorescein release (g^{-1} substrate); FvFm – photosynthetic efficiency; Height – plant height; Leaf_area – total leaf area per plant; Nematodes – number of nematode eggs per plant; Photo – photosynthesis rate; Rf – nematode reproduction factor; Root_weight – fresh root weight; Trans – transpiration; and Yield – effective quantum yield of PSII. The data projected on the first two PCA axes explain most of the observed variance. Sample principle component scores are shown as dots coloured according to the treatments, while variables are shown as vectors plotted within the correlation circle. All variables were min-max normalised to 0-1 range prior to analysis. Samples were additionally assigned to clusters (dotted lines) based on the results of hierarchical cluster analysis. NC – negative control; PC – positive control (nematicide); BfL – Low *B. firmus* inoculum; BfH – High *B. firmus* inoculum.

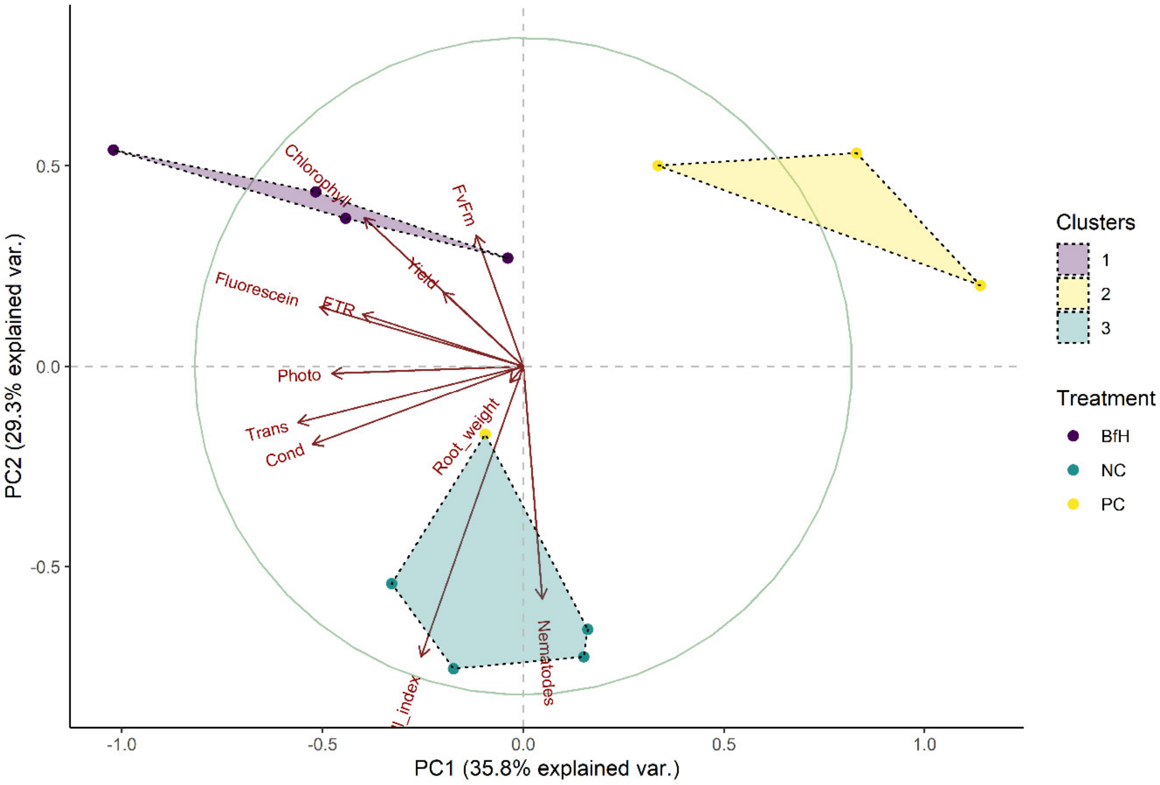

Figure S2. Principal component analysis (PCA) biplot of the microplot experiment data, based on 11 variables measured in 12 plants across the NC, PC and BfH treatments. The variables used in PCA were: Chlorophyll – relative chlorophyll content; Cond – stomatal conductance; ETR – electron transport rate; Fluorescein – fluorescein release (g^{-1} soil); FvFm – photosynthetic efficiency; Gall_index – nematode galling index; Nematodes – number of nematodes (g^{-1} soil); Photo – photosynthesis rate; Root_weight – fresh root weight; Trans – transpiration; and Yield – effective quantum yield of PSII. The data projected on the first two PCA axes explain most of the observed variance. Sample principle component scores are shown as dots coloured according to the treatments, while variables are shown as vectors plotted within the correlation circle. All variables were min-max normalised to 0–1 range prior to analysis. Samples were additionally assigned to clusters (dotted lines) based on the results of hierarchical cluster analysis. NC – negative control; PC – positive control (nematicide); BfH – High *B. firmus* inoculum.

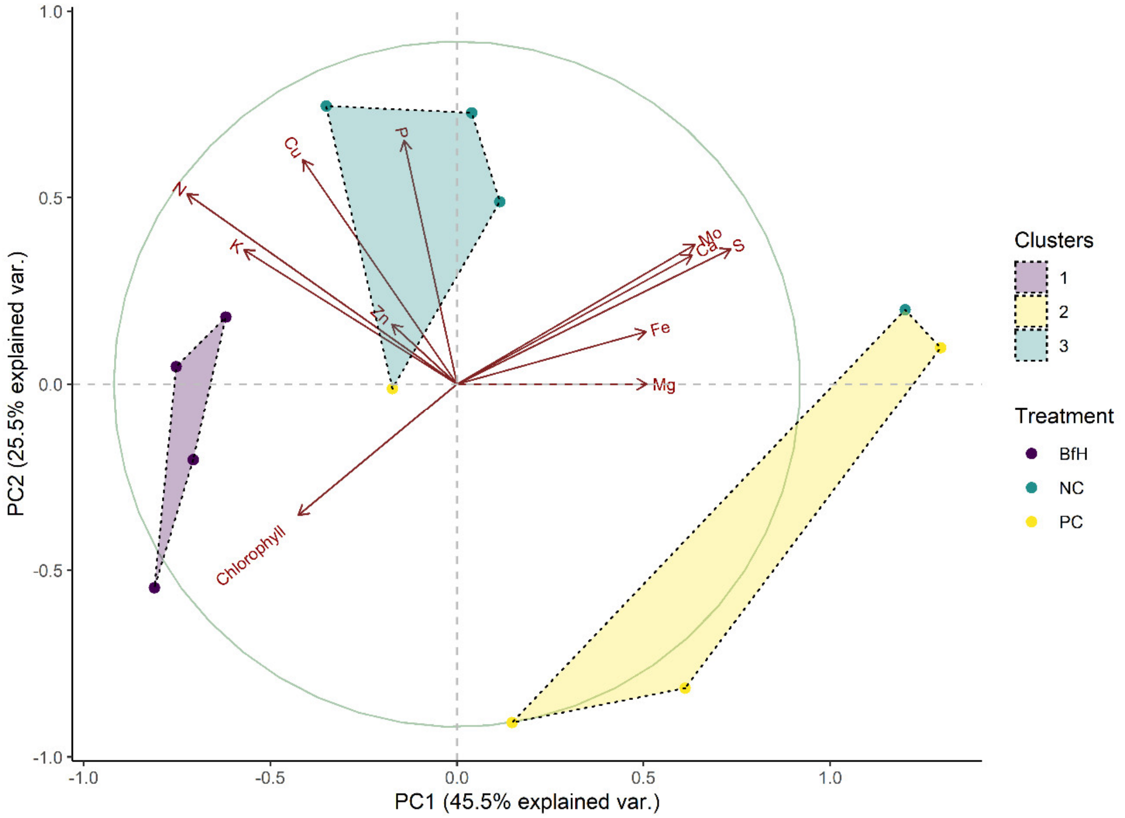

Figure S3. Principal component analysis (PCA) biplot of the microplot experiment data, based on 11 nutrients measured in 12 plants across the NC, PC and BfH treatments. The variables (nutrient content in leaf tissue) used in PCA were: Ca – calcium; Chlorophyll – relative chlorophyll content; Cu – copper; Fe – iron; K – potassium; Mg – magnesium; Mo – molybdenum; N – nitrogen; P – phosphorous; S – sulphur; and Zn – zinc. The data projected on the first two PCA axes explain most of the observed variance. Sample principle component scores are shown as dots coloured according to the treatments, while the variables are shown as vectors plotted within the correlation circle. All variables were min-max normalised to 0–1 range prior to analysis. Samples were additionally assigned to clusters (dotted lines) based on the results of hierarchical cluster analysis. NC – negative control; PC – positive control (nematicide); BfH – High *B. firmus* inoculum.

References

1. RStudio Team *RStudio: Integrated Development for R*; RStudio, Inc.: Boston, Massachusetts, USA, 2016. Available online: http://www.rstudio.com/ (accessed on 18 February 2020).
2. Vu, V. *ggbiplot: An implementation of the biplot using ggplot2*; Columbus, Ohio, 2015. Available online: https://github.com/vqv/ggbiplot (accessed on 18 February 2020).
3. Oksanen, J.; Blanchet, F.G.; Friendly, M.; Kindt, R.; Legendre, P.; McGlinn, D.; Minchin, P.R.; O'Hara, R.B.; Simpson, G.L.; Solymos, P.; et al. *vegan: Community Ecology Package*; 2019. Available online: https://CRAN.R-project.org/package=vegan (accessed on 18 February 2020).

© 2020 by the authors. Submitted for possible open access publication under the terms and conditions of the Creative Commons Attribution (CC BY) license (http://creativecommons.org/licenses/by/4.0/).
